# Biochar from co-pyrolysis of biological sludge and woody waste followed by chemical and thermal activation: end-of-waste procedure for sludge management and biochar sorption efficiency for anionic and cationic dyes

**DOI:** 10.1007/s11356-024-33577-3

**Published:** 2024-05-09

**Authors:** Zaineb Bakari, Michelangelo Fichera, Ayoub El Ghadraoui, Lapo Renai, Walter Giurlani, Daniela Santianni, Donatella Fibbi, Maria Concetta Bruzzoniti, Massimo Del Bubba

**Affiliations:** 1 Department of Chemistry “U. Schiff”, University of Florence, Via Della Lastruccia 3, 50019 Florence, Sesto Fiorentino Italy; 2 Laboratory of Environmental Engineering and Ecotechnology (LR16ES19), National Engineering School of Sfax, Route de La Soukra Km 4, 3038 Sfax, Tunisia; 3 Publiacqua S.P.A, Via Villamagna 90 C, 50126 Florence, Italy; 4 Gestione Impianti di Depurazione Acque (G.I.D.A.) S.P.A, Via di Baciacavallo 36, 59100 Prato, Italy; 5 Department of Chemistry, University of Turin, Via Pietro Giuria 7, 10125 Turin, Italy

**Keywords:** Industrial wastewater remediation, Design of experiment, Biochar production, Biochar characterization, Methylene blue, Direct yellow 50, Isotherm experiments, Column experiments

## Abstract

Nine biochars were produced by co-pyrolysis of sawdust and biological sludge following the “design of experiment” approach. Two kinds of sludge (both deriving from the treatment of mixed industrial-municipal wastewater) and two types of woody waste were selected as categorical predicting variables, while contact time, pyrolysis temperature, and sludge percentage were used as quantitative variables. Biochars were analysed for their product characteristics and environmental compatibility based on the European Standards (EN 12915–1:2009) for materials intended for water treatment (i.e. ash content, water leachable polycyclic aromatic hydrocarbons (PAHs) and elements), as well as for specific surface area (SSA), using them as response variables of a multivariate partial least square multiple regression, whose results provided interesting insights on the relationships between pyrolysis conditions and biochar characteristics. Biochars produced with sludge and/or providing the highest SSA values (258–370 m^2^ g^−1^) were selected to undergo a sustainable chemical treatment using a by-product of the gasification of woody biomass, complying in all cases with European Standards and achieving therefore the end-of-waste status for sewage sludge. The biochar deriving from the highest percentage of sludge (30% by weight) and with the highest SSA (390 m^2^ g^−1^) was thermally activated achieving SSA of 460 m^2^ g^−1^ and then tested for the sorption of direct yellow 50 and methylene blue in ultrapure water and real wastewater, compared to a commercial activated carbon (AC). The biochar showed Langmuir sorption maxima (*Q*_m_) 2–9 times lower than AC, thus highlighting promising sorption performances. *Q*_m_ for methylene blue in wastewater (28 mg‧g^−1^) was confirmed by column breakthrough experiments.

**Supplementary Information:**

The online version contains supplementary material available at 10.1007/s11356-024-33577-3.

## Introduction

The quality of surface water (SW), as well as that of wastewater treated for reuse, is increasingly compromised by the presence of organic micropollutants of both domestic and industrial origin. Within this latter category of micropollutants, synthetic dyes are structurally heterogeneous compounds widely used in many manufacturing applications, mainly related to textile industry (Sabnis 2017). Synthetic dyes are generally characterized by a significant toxicity towards humans and environment, which is expressed in most cases through their degradation products, rather than by the parent molecule. For example, the toxicity of azo-dyes occurs through the reduction and cleavage of the azo linkage to give aromatic amines that are metabolically oxidized to reactive electrophilic species, capable to covalently bind DNA, or via direct oxidation of the azo group to highly reactive electrophilic diazonium salts (Brown and Vito 1993). Accordingly, adsorption techniques may play an important role in the removal of dyes from wastewater since they do not provide any modification of the structure of the molecule during the treatment.

Both well-established materials (i.e. commercial activated carbons) (Azari et al. 2020) and novel sorbents (e.g. polymer-derived silicon carbide foams and aerogels and various kinds of nanocomposites) (Bruzzoniti et al. 2018a, 2018b; Srivastava et al. 2020) have been tested for the removal of dyes from water matrices. Among sorbents intended for wastewater treatment, biochar has received increasing attention as a low-cost material obtained from the thermochemical conversion (i.e. pyrolysis or gasification) of a wide variety of waste vegetal biomass and/or biosolids (Ghodake et al. 2021). Biochar is a porous material containing crystalline (i.e. graphene-like) and amorphous carbonized fractions, as well as non-carbonized phases, which may comprise aromatic and aliphatic portions linked with groups at different polarity (Inyang &Dickenson 2015). According to literature, biochars exhibit a wide range of physical and chemical characteristics, depending on the thermochemical conversion process, the temperature, the contact time, and the feedstock used for their production (Castiglioni et al. 2021). Moreover, various modifications and/or engineering of biochars (e.g. chemical and physical activation, metal impregnation, and functionalization) have been tested in order to enhance their sorption efficiency towards micropollutants in water matrices (Liu et al. 2015; Tan et al. 2017). Among the waste biomass used as feedstock for biochar production, biological sludge from wastewater treatment plants (WWTPs) represents an attractive option. Biological sludge is in fact a widely available waste material characterized by a high environmental hazard, the disposal of which involves a high cost, while its reuse is consistent with the modern approach to the waste management and the circular economy (European Parliament and the Council of the European Union 2018), thereby ensuring great savings. It is also noteworthy that thermochemical conversion of biological sludge into biochar, which takes place at temperatures above about 400 °C, allows to inhibit the intrinsic toxicity linked to the presence in this matrix of poorly biodegradable organic micropollutants and potentially pathogenic organisms (i.e. viruses and bacteria). Therefore, it is clear that the conversion of biological sludge into biochar to be used as sorbent in WWTPs may be a doubly successful strategy to achieve more efficient sludge management and better environment protection. The use of biological sludge, alone (Singh et al. 2020) or mixed with waste vegetal biomass (Chen et al. 2019; Zhang et al. 2020a), has recently been investigated for the production of biochar in some researches. Nevertheless, the thermal conversion of biological sludge from industrial wastewater treatment to biochar is poorly described in the literature (Kwon et al. 2020). Sludge-based biochars usually exhibit values of specific surface area (SSA) and sorption properties much lower than commercial activated carbons (Castiglioni et al. 2021), the latter considered the material of choice for the removal of micropollutants in wastewater and drinking water (Del Bubba et al. 2020). Hence, biochars are often modified and/or engineered following different approaches (e.g. chemical and physical activation, metal impregnation, and synthesis of biochar-derived nanocomposites), in order to enhance their adsorption properties (Cheng et al. 2021). However, engineered biochars have their own limitations, such as the consumption and/or release of chemicals or nanomaterials potentially hazardous for the environment, as well as higher costs compared to those obtained by thermal conversion only (Lyu et al. 2018; Wan et al. 2019).

Sludge-based biochars have been tested for the adsorption and the degradation of various micropollutants, mainly metal cations, pharmaceuticals, and dying agents (Singh et al. 2020). However, these studies were performed in most cases on ultrapure water solutions, thus limiting their significance. Moreover, the removal performance of biochars is seldom compared with the adsorption capacity of standard activated carbons (Castiglioni et al. 2022; Del Bubba et al. 2020), with obvious limitations in the reliable evaluation of their sorption performance.

Although to our knowledge there is no legislation regulating the use of biochar and more generally of adsorbent materials in the treatment of wastewater, the EN 12915–1 European Standards regulating the use of adsorbent materials for the treatment of drinking water (European Committee for Standardization 2009) can be used as a precautionary approach. This regulation provides legal limits for parameters related to adsorption performances (e.g. ash content) and environmental compatibility (i.e. release of selected metals and polycyclic aromatic hydrocarbons), the latter commonly representing a relevant issue in the use of biochars but very often not evaluated in the literature (Singh et al. 2020).

Based on the above-mentioned considerations, the objective of this research was the production and characterization of biochars by co-pyrolysis of mixtures of biological sludge (both from the treatment of mixed industrial-municipal wastewater) and waste woody biomass, under different experimental conditions, set by the experimental design approach (DoE). The biochars have been characterized for some product and environmental compatibility parameters included in the aforementioned European Standards, here considered a precautionary guideline, thus providing new information on the end-of-waste process of biological sludge. These parameters were used in DoE as response variables of a multivariate partial least square multiple regression (PLS). Biochars providing the highest SSA values were treated by chemical and thermal processes to improve their product and environmental compatibility properties and finally characterized in depth. Sorption capacity towards methylene blue (MB) and direct yellow 50 (DY) was determined for biochars better satisfying the European Standards requirements, in comparison with a commercially available virgin activated carbon (AC), commonly employed in WWTPs and potabilization facilities. Ultrapure water and effluent wastewater, the latter collected from a WWTP operating in an industrial textile district, were used for this purpose. In particular, the wastewater used in this study derived from one of the two WWTPs from which the sludge was extracted, allowing to collect new information about the sludge management cycle within this kind of WWTPs. Sorption performances were discussed in relation to physicochemical properties of the materials in order to interpret the removal data obtained. Breakthrough column tests were also carried out with the material having the most promising performance to evaluate MB removal from wastewater.

## Materials and methods

Full details of the reagents, standards, and materials used in this research, as well as preparation of stock solutions of target analytes, are reported in section S.1 of the *Supplementary material*, together with CAS numbers, structure formulas, log *K*_OW_ values, and maximum absorption wavelengths of the investigated dyes (see Table S1). Target analytes are characterized by different charges (− 4 and + 1 for DY and MB, respectively, at pH = 7) and hydrophobicity (log *K*_OW_ values at pH = 7, equal to − 1.85 and 2.61 for DY and MB, respectively).

### Feedstocks used for biochar production

The biological sludge (BSs) and the waste woody biomass (WWBs) used as feedstocks for the production of biochar were supplied by Gestione Impianti di Depurazione Acque S.p.A. (Prato, Italy) and Romana Maceri Centro Italia S.r.l. (Civitella in Val di Chiana, Italy), respectively. More in detail, WWBs were residues from the cutting of two forests in Tuscany (Italy), mainly consisting of oak and poplar, respectively, while BSs derived from Calice and Vernio activated sludge WWTPs (see Table S2, section S.2 of the *Supplementary material* for their characterization), mainly treating industrial textile wastewater. The feedstocks were dried at 90 °C for 48 h and then crushed and sieved at 10 mm before being pyrolysed.

### Biochar and activated carbons

The biochars were produced via co-pyrolysis of WWBs and BSs mixtures in a muffle furnace (Gefran 1001, Vittadini Strumentazione, Milano, Italy), properly modified to allow the heating process in N_2_-saturated conditions. All materials were pre-treated before being characterized and used in isotherm studies. In detail, the chars were sieved at 45 µm and then repeatedly washed with ultrapure water according to the ASTM D-5919–96 method (American Society for Testing and Material 2022).

### Design of experiments

The biochars were produced following the DoE approach, through a “reduced combinatorial design” that allowed to balance the number of experiments with the efforts required for the analysis of the multiple parameters, necessary for an in-depth characterization of the sorbents. In this way, it was possible to treat equally qualitative (feedstock and sludge type) and multilevel quantitative (i.e. pyrolysis temperature, contact time, sludge percentage) factors. According to this design, nine biochars (B1–B9) plus three replicates (B3/B10, B8/B12, and B9/B11) were produced under the experimental conditions reported in Table 1. The aforementioned factors were used for modelling some relevant parameters related to product characteristics (i.e. SSA and ash content) and environmental compatibility (i.e. released of PAHs and selected elements) of the biochars, through the PLS algorithm. Correlation coefficients in fitting (*R*^2^) and prediction (*Q*^2^) ≥ 0.50 were applied as constraints to consider statistically significant the models obtained. The software Sartorius (Stedim Biotech, Aubagne, France) MODDE Pro 13.0 was used for planning the data processing related to the “reduced combinatorial design” and the evaluation of the PLS models.

**Table 1:** Matrix of the production experiments of biochars and mean values of yields (*n* = 12, standard deviation in bracket) obtained as percentages of the dried feedstock. Biochars marked with the same superscript letter are samples referring to different productions under the same experimental conditions. Woody waste was mainly oak (A) or poplar (B)

Biochar	Woody waste	Sludge type	Temperature (°C)	Contact time (min)	Sludge percentage	Yield
B1	A	-	450	120	0	31 (1)
B2	A	-	650	60	0	20 (4)
B3^(a)^	B	-	850	120	0	18 (2)
B4	B	Calice	450	60	15	34 (4)
B5	A	Vernio	650	120	15	30 (1)
B6	B	Vernio	850	60	15	31 (1)
B7	A	Vernio	450	60	30	37 (2)
B8^(b)^	B	Calice	650	120	30	31 (6)
B9^(c)^	A	Calice	850	60	30	34 (8)
B10^(a)^	B	-	850	120	0	14 (4)
B11^(c)^	A	Calice	850	60	30	39 (7)
B12^(b)^	B	Calice	650	120	30	29 (6)

### Char characterization

#### Elemental analysis

The C, H, N, S analysis was performed using a FlashEA® 1112 elemental analyser Thermo Fisher Scientific (Waltham, MA) equipped with a thermal conductivity detector (Table S3 of the *Supplementary material*). The percentage content of oxygen was estimated as the difference with those of the other elements and ash (Al-Wabel et al. 2013). Moreover, the O/C and H/C ratios were calculated and plotted in a van Krevelen diagram (Figure S1 of the *Supplementary material*). Full details of the elemental analysis and related calculations are reported in section S.3.1 of the *Supplementary material*.

#### Ash

Ash content was determined according to the EN 12902:2004 Official Method (European Committee for Standardization 2004), which refers to the analysis of products used for the treatment of water intended for human consumption and is adopted in this field for the analysis of activated carbons. Full details of the procedure adopted for the determination of ash content and results obtained are respectively reported in section S.3.2 and Table S4 of the *Supplementary material*.

#### Physisorption analysis

Physisorption analysis of biochars was performed via nitrogen adsorption and desorption experiments using a Porosity Analyser Micrometrics (Norcross, GA, USA) model 3Flex (American Society for Testing and Material 2022). Total SSA, as well as micropore and mesopore SSA, was determined by the Brunauer–Emmett–Teller (BET), the t-plot, and the Barrett-Joyner-Halenda (BJH) methods, respectively (see section S.3.3 of the *Supplementary material* for further details).

#### pH of the point of zero charge

The pH of the point of zero charge (pH_PZC_) was determined using the pH drift method, which is a simple and widely used procedure, adopted for the evaluation of the surface charge of both biochars (Chaukura et al. 2017) and activated carbons (Niasar et al. 2016). Full details of the procedure are reported in section S.3.4 of the *Supplementary material*, together with the plots obtained for the investigated biochars (Figure S2).

#### Thermogravimetric analysis

Thermogravimetric analysis (TGA) was conducted using a TGA analyser, model EXSTAR 6200, as specified in section S.3.5 of the *Supplementary material*.

#### X-ray diffraction analysis

X-ray diffraction (XRD) was performed by using the Bruker (Billerica, MA, USA) New D8 Da Vinci X-ray diffractometer (radiation Cu-Kα1 = 1.54056 Å, 40 kV × 40 mA) equipped with a Bruker LYNXEYE-XE detector (see section S.3.6 of the *Supplementary material* for further details).

#### Scanning electron microscopy-energy dispersive X-ray spectroscopy

Scanning electron microscopy (SEM) and energy dispersive X-ray analysis (EDX) were performed by a Hitachi (Tokyo, Japan) SU3800 SEM equipped with a Silicon Drift EDS Detector, model Ultim Max 40 (Oxford Instruments NanoAnalysis, High Wycombe, UK). For further details, see section S.3.7 of the *Supplementary material*.

#### Water-extractable substances

The aqueous extraction of selected elements and PAHs (Table S4) was performed according to the EN 12915–1 standard method (European Committee for Standardization 2009), as described in section S.3.8 of the *Supplementary material*. The analysis of the water-extractable metals was carried out by ICP-MS after microwave digestion, while PAHs were determined by SPE extraction followed by GC–MS analysis. Full details of both analytical protocols and figures of merit are reported in Tables S5-S7 and Figure S3 (section S.3.8 of the *Supplementary material*).

### Effluent wastewater used in sorption and column studies

Effluent wastewater from the Calice WWTP was used to evaluate the sorption capacity of the biochar, which has proven to be more efficient in tests with ultrapure water. The characterization of the effluent wastewater for a number of routinely analysed parameters is shown in Table S8 (section S.4 of the *Supplementary material*).

#### Chemical and thermal activation of biochars

The biochars were treated with the BioDea® solution, a commercially available (Bio-Esperia S.r.l., Umbertide, Italy) acidic liquid by-product of the gasification of woody waste (see section S.5 of the *Supplementary material* for some details of the optimization of the washing protocol and Table S9 for BioDea characteristics) and then thermally activated at 650 °C for 2 h in muffle furnace under N_2_-saturated conditions. These experimental conditions were chosen based on the results obtained in previous studies for the regeneration of chars from coconut shell (Cazetta et al. 2013). Activated biochars were dried overnight at 105 °C and kept in a desiccator before use.

### Performance evaluation of selected chars as adsorbent materials

The evaluation of the adsorption performances of biochars and AC towards the investigated dyeing agents was carried out by means of kinetic and isotherm tests, measuring the decrease of the absorbance of aqueous solutions of MB (652 nm) and DY (404 nm) in contact with chars by an UV–Vis spectrophotometer HACH (Loveland, CO, USA) DR/4000 U. Full details of the procedures adopted for carrying out these tests are reported in section S.6 of the *Supplementary material*.

Langmuir and Freundlich equations were used to fit the adsorption isotherms data, as described in section S.6.2 of the *Supplementary material*.

Breakthrough column tests were also carried out as described in section S.6.3 in order to test the sorption capacity of biochar under experimental conditions closer to those adopted in WWTPs for wastewater refining by filtration on activated carbons.

### Data treatment and computational analysis

The construction of the linearized Langmuir and Freundlich models and the evaluation of their statistical significance through the Fisher test was carried out using Microsoft Excel® 2019 (Redmond, WA, USA).

The molecular width of target analytes was calculated after MM2 energy minimization using the Chem3D package, version 12.0.2.1076 (PerkinElmer Informatics, Waltham, MA, USA).

## Results and discussion

### Evaluation of the biochar characteristics in relation to PLS models

**Fig. 1:**
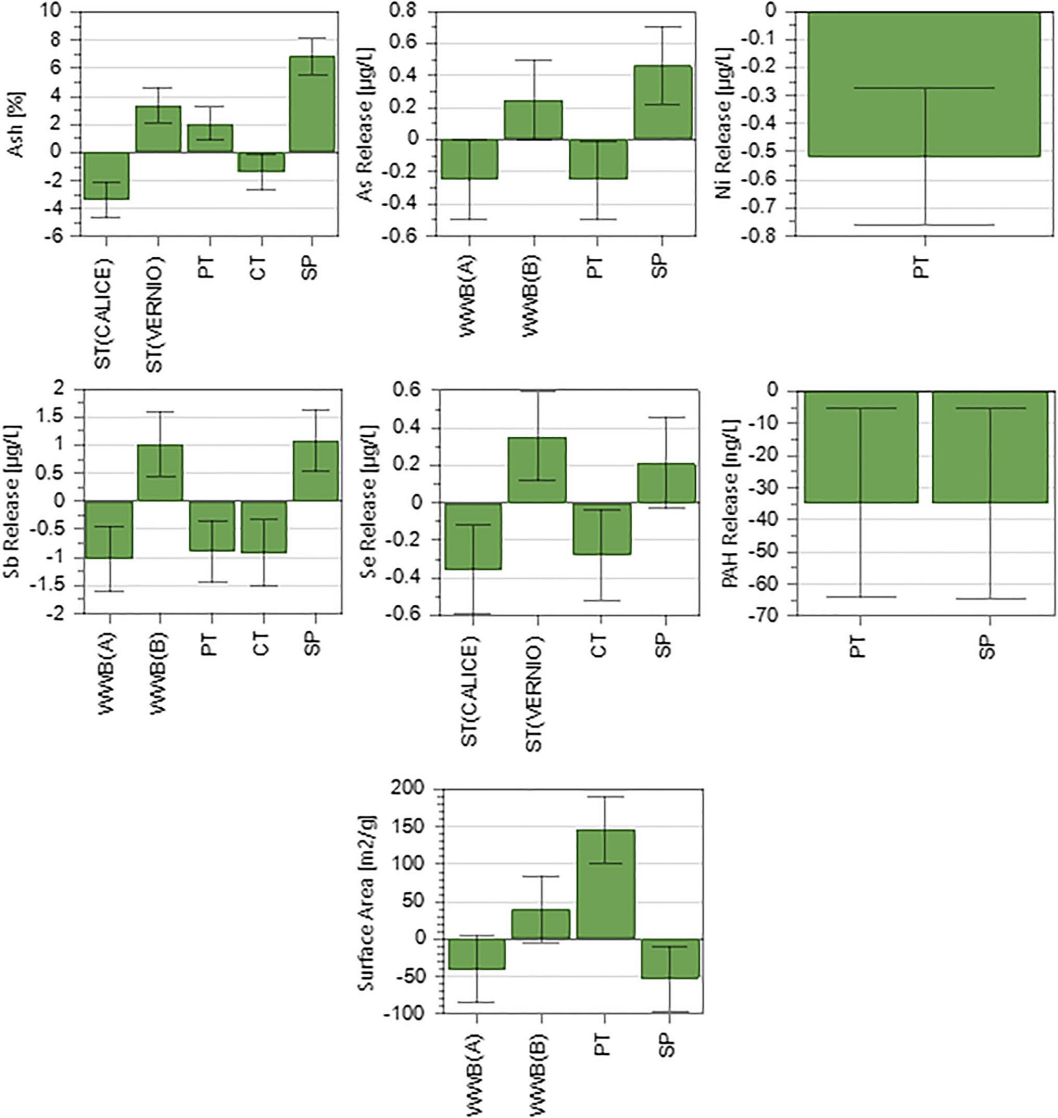
Scaled and centred coefficients of the statistically significant partial least square regression models for the best fitting and prediction of ash content, release of As, Ni, Sb, Se, and PAHs, and specific surface area, based on the following production factors: woody waste type (WWB), sludge type (ST), pyrolysis temperature (PT), contact time (CT), and sludge percentage (SP). WT was mainly oak (A) or poplar (B)

Biochars produced under the experimental conditions described in the screening matrix (Table 1) have been characterized for ash content and water extractable trace elements and PAHs (Table S4), as parameters regulated by the EN 12915–1 for materials intended for water filtration. Moreover, the total SSA of the investigated materials has also been determined (Table S4) since it provides interesting information on the sorption ability (Luo et al. 2022). These parameters have been used as response variables in the experiments included in the screening matrix. Values of pH_PZC_ and distribution of the main elements (C, H, N, S, and O) (Table S3 of the *Supplementary material*) were also determined on all biochars reported in Table 1, and van Krevelen diagram (H/C vs. O/C) was plotted (Figure S1 of the *Supplementary material*) in order to obtain general information about composition and stability of the biochars produced. However, these data were not included in the group of response variables, since they are not directly related to the environmental compatibility or sorption capacity of carbonaceous materials. Based on the constraints adopted for *R*^2^ and *Q*^2^ values (see Table S10, section S.7 of the *Supplementary material*), statistically significant PLS models were obtained for ash content, As, Ni, Sb, Se, and PAHs release, and SSA. Figure 1 illustrates the scaled and centred PLS model coefficients associated with the multilevel and qualitative production factors (prediction variables), which exerted a statistically significant effect on the aforementioned seven response variables. Considering the values assumed by these coefficients, it is possible to infer their influence on the response variables.

#### Ash

Higher values of sludge percentage (SP) contribute to an increase in the ash content of biochars, even though with different extents by the two sludge types (ST), being the Calice and Vernio coefficients characterized by opposite signs. This finding reflects the high content of inert constituents of sewage sludge used here, particularly that deriving from the Vernio WWTP (see Table S2). The ash content also increases with increasing pyrolysis temperature (PT), in accordance with the variations of the yield of biochar reported elsewhere (Castiglioni et al. 2022) as a function of this production factor. Conversely, contact time (CT) shows an opposite influence, even though the extent of its contribution is quite low.

#### Selected elements

Similar to what has been observed for ash, the release of selected elements (i.e. As, Sb, and Se) increases with the percentage of sludge. Moreover, the two types of vegetal biomass and the two sludges showed different contributions, as evidenced by the opposite signs of their coefficients, thus suggesting a lower metal bioavailability for biochar made from oak and sludge of Calice WWTP, compared to those produced from poplar and sludge of Vernio WWTP. The release of elements is also affected by PT (for As, Ni, and Sb) and CT (for Sb and Se), both exhibiting negative coefficients and thus highlighting a lower water extractability in biochar produced at higher PT and CT. Similar results have been found in previously published studies (Devi & Saroha 2014; Zhang et al. 2022) and explained on the basis of complex phenomena occurring in the material, which involve the decomposition of organic functional groups, the collapse of the pores, and the incorporation of the inorganic species into the new structure formed (Zhang et al. 2022). Further possible explanations involve the presence/formation of salts and crystalline phases having basic character that bind the elements, reducing their leachability (Devi &Saroha 2014). This hypothesis is strongly supported by the much higher pH_PZC_ values found for biochars obtained at 650–850 °C, compared to those produced at 450 °C (Table S3).

#### Polycyclic aromatic hydrocarbons

PT and SP significantly affect the release of PAHs, which decreases with the increase of both these parameters. The effect of PT observed here confirms the results elsewhere reported and attributed to the degradation of PAHs already present in the sludge and/or formed during pyrolysis at temperatures up to about 500 °C (Castiglioni et al. 2022). The decreasing trend of the release of PAHs with the increase of SP can be explained by the different compositions of the organic fractions of WWB and sludge. In fact, the woody biomass consists almost exclusively of cellulose, hemicellulose, and lignin, well-known precursors of the formation of PAHs during pyrolysis (Wang et al. 2017), while the bacterial biomass, made mainly of peptidoglycans and lipids, is less subjected to PAH formation (McGrath et al. 2007). The decrease in PAHs release as SP in the feedstock increases is a very interesting result that highlights a further positive aspect of the management of sludge by pyrolysis.

#### Specific surface area

The feedstock composition and the PT significantly influenced the surface area. In detail, the increase in SP negatively affects the surface area regardless of the ST. Conversely, WWB exerts a different effect depending on the type of biomass, with WWB-A (mainly oak) and WWB-B (mainly poplar) contributing to lower and increased surface area, respectively. Surface area increases by increasing PT, in agreement with findings previously obtained on various types of biochars (Castiglioni et al. 2022).

#### Overall evaluation of pyrolysis conditions

Overall, based on the coefficients reported in Fig. 1, it is evident that PT and/or SP significantly affected all the seven response variables modelled by PLS. Therefore, it is interesting to evaluate the trend of these response variables as a function of both SP and PT, attempting an optimization of the pyrolysis process. This can be done through the 2D contour plots reported in Figure S4-S7 of the *Supplementary material*, where, in each of the four plots, a combination of the categorical variables WWB and ST is selected, while the multilevel factor CT is fixed at 90 min, owing to its lower statistical inference, compared to the other quantitative variables. In these plots, target values represent the EN 12915–1 requirements reported in Table S4, except for SSA which was fixed at 300 m^2^ g^−1^ based on an overview of literature data (El Barkaoui et al. 2023). The examination of the contour plots highlights that in the investigated ranges of the process variables; the behaviour of the seven response variables modelled by PLS is identical for all four possible combinations of feedstocks subjected to co-pyrolysis. Changes in process conditions that improve certain product and/or environmental characteristics of the biochars lead to a worsening of others. For example, when producing biochar at high temperatures and with high sludge percentages, the production and release of PAHs are minimized, but at the same time, ash percentages much higher than the target value of 15% are obtained. It should also be noted that within the regulatory group of elements, chromium (for which statistically significant PLS models could not be identified) was in all cases above the limit (Table S4).

### Post-synthesis chemical treatment and thermal activation

The PLS results presented above clearly indicate the need for further treatments to improve the precautionary parameters foreseen by the European Standards. For this purpose, a sustainable chemical treatment strategy has been developed using the BioDea solution, a by-product of the gasification of wood waste biomass for energy production. This solution is characterized by a high content of acetic acid and phenolic acids with a distinctly acidic pH (see Table S9 for its characterization), thus being, in principle, suitable for the removal of inorganic species from the produced biochars.

In detail, B5, B6, B8-B12, and B9-B11 (i.e. the mixture of the biochars obtained with different production batches made under the same experimental conditions) were selected for the BioDea treatment as the materials produced with all the possible combinations of ST and WWB (Table 1) and providing the highest SSA values (258–370 m^2^ g^−1^). These values are higher than those of biochars obtained elsewhere by co-pyrolysis of biological sludge with a number of vegetal feedstocks, such as bamboo sawdust (3–8 m^2^ g^−1^), rice husk (4–11 m^2^ g^−1^) (Zhang et al. 2020b), and wheat straw (75–267 m^2^ g^−1^) (Deng et al. 2017). B3-B10 was also chosen as the biochar replicates produced only from WWB and exhibiting the highest SSA values (552–580 m^2^ g^−1^).

**Fig. 2:**
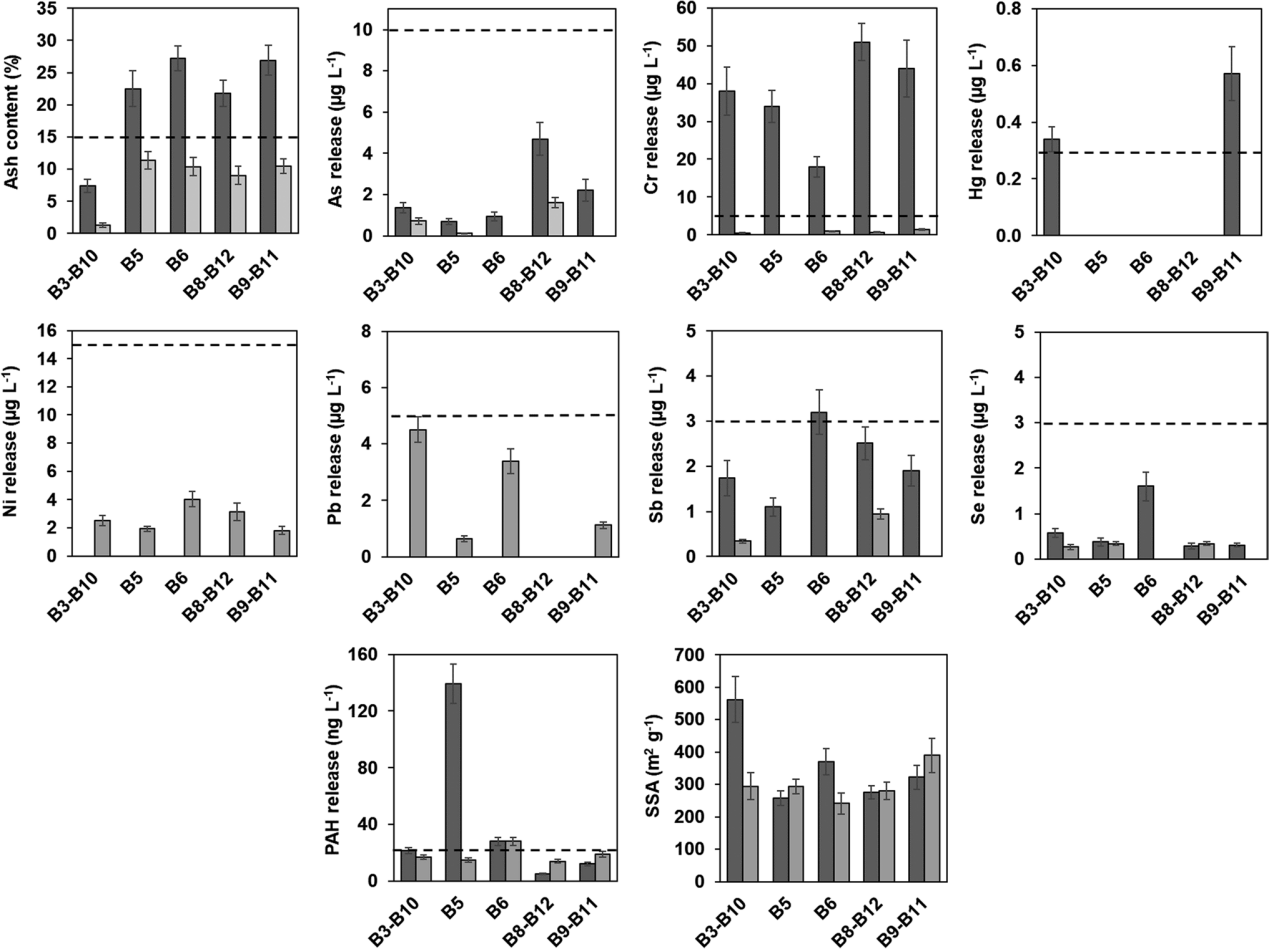
Mean values (*n* = 3) and standard deviation of ash, water extractable elements and polycyclic aromatic hydrocarbons (PAHs), and specific surface area (SSA) determined in biochars as such (dark grey bars) and treated with the BioDea solution (light grey bars). The dashed lines indicate the limits established by the UNI EN 12915–1. B3-B10, B8-B12, and B9-B11 refer to samples obtained by mixing equal aliquots of the two biochars

Figure 2 illustrates the effects of the BioDea treatment on the ash content, elements and PAHs release, and SSA of the aforementioned biochars. This treatment was able to strongly lower the ash content of all the materials investigated, even reducing it below the precautionary limit of 15% required by EN 12915–1. A similar result was obtained also for most of the leachable elements covered by this regulation, in accordance with the highly acidic and complexing characteristics of the BioDea solution, ascribable to the presence of high concentrations of acetic acid and (poly)phenolic substances (see Table S9). Pb and Ni represent exceptions as their leachable concentration before treatment was found to be below the quantification limit for all selected materials (i.e. < 0.5 µg L^−1^), while after washing with BioDea, the leachate showed quantifiable concentrations of both metals, albeit very low and within the limits of the aforementioned standard. In this regard, it should be noted that Ni and Pb were by far the most abundant elements found in BioDea among those included in EN 12915–1. Hence, it can be hypothesized that these metals were weakly adsorbed onto the materials during the washing procedure with BioDea and then released with the leaching test, which is performed with an aqueous solution containing significant concentrations of Na, Ca, and Mg ions (see section S.3.8 of the *Supplementary material*). The treatment with BioDea and subsequent washing with deionized water did not produce appreciable variations in the release of PAHs from most biochars (Fig. 2), being B5 the sole exception. This biochar was the only material with leachable PAHs concentrations before treatment much higher than the limit of the European standard and showed a sharp reduction of such concentrations after treatment.

Overall, the results of the leaching test after the treatment with BioDea are interesting since they highlight the absence of environmental drawbacks in the use of this chemical treatment, notwithstanding its nature of “secondary product” obtained within the gasification of waste vegetal biomass for energy production.

**Fig. 3:**
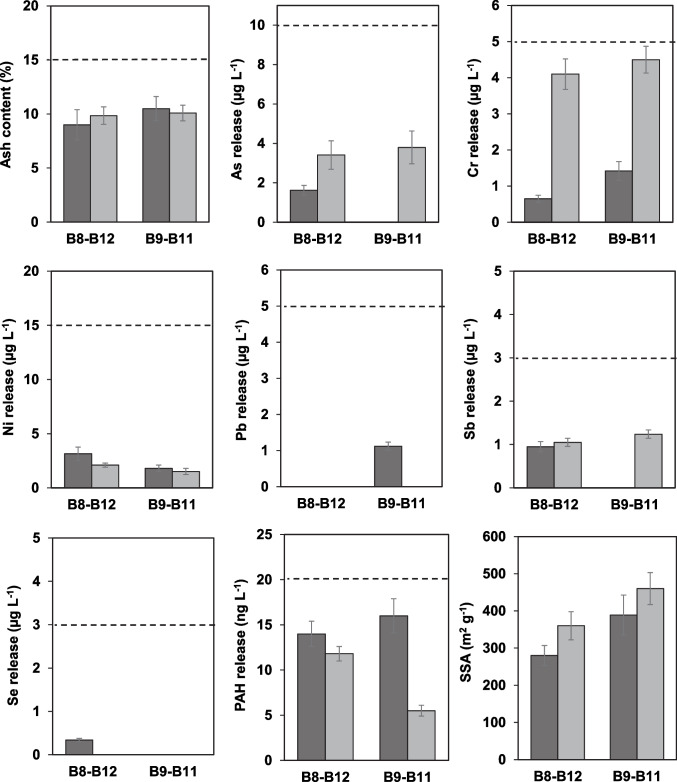
Mean values (*n* = 3) and standard deviation of ash, water extractable elements and polycyclic aromatic hydrocarbons (PAHs), and specific surface area (SSA) determined in biochars after treatment with the BioDea solution (dark grey bars) and successive thermal activation (light grey bars). The dashed lines indicate the limits established by the UNI EN 12915–1. B8-B12 and B9-B11 refer to samples obtained by mixing equal aliquots of the two biochars

Although the treatment with BioDea was able to remove significant amounts of inert constituents from the biochars, thus suggesting the possible increase of their porosity, no significant changes in SSA were observed in B5, B8-B12, and B9-B11, while there was even a significant decrease in the surface area of B6 and B3-B10. Thermal activation of B8-B12 and B9-B11 was then performed in an attempt to improve the SSA values. After this treatment, all the investigated leachable pollutants remained within the European standards, with main changes represented by the increase of Cr and the reduction of PAHs in the leachate of both treated materials (Fig. 3). However, the main result produced by the thermal activation was the higher SSA, which reached mean values of 360 and 460 m^2^ g^−1^ for B8-B12 and B9-B11, respectively, corresponding to an increase of about 20%, compared to the not-activated materials. These values are remarkably interesting, considering that the biochars were prepared from a 30/70 dw/dw biological sludge-woody waste mixture and that the available SSA values for biochars derived from similar feedstocks are lower than those determined in this study (see Table S11, Section S.8 of the *Supplementary material*).

**Fig. 4:**
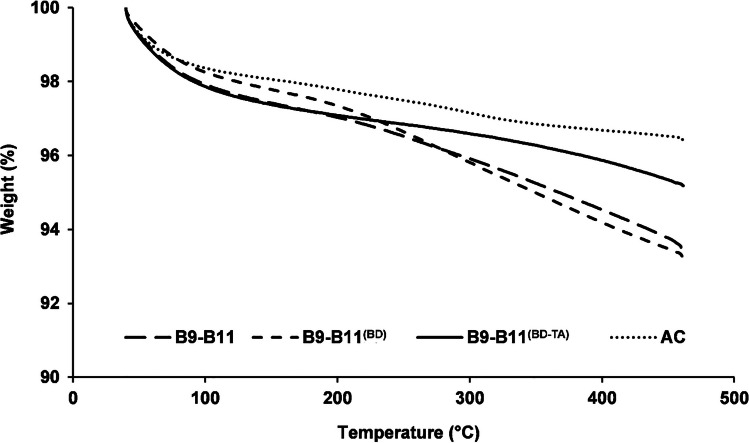
Thermogravimetric analysis of the biochar B9-B11 (wide dashed line), after washing with BioDea (B9-B11^(BD)^, dashed line), and successive thermal activation (B9-B11^(BD−TA)^), solid line), in comparison with a commercial activated carbon (AC, dotted line). The analysis was performed from 40 to 460 °C at 10 °C min^−1^ under nitrogen flow of 100 mL min^−1^

**Fig. 5:**
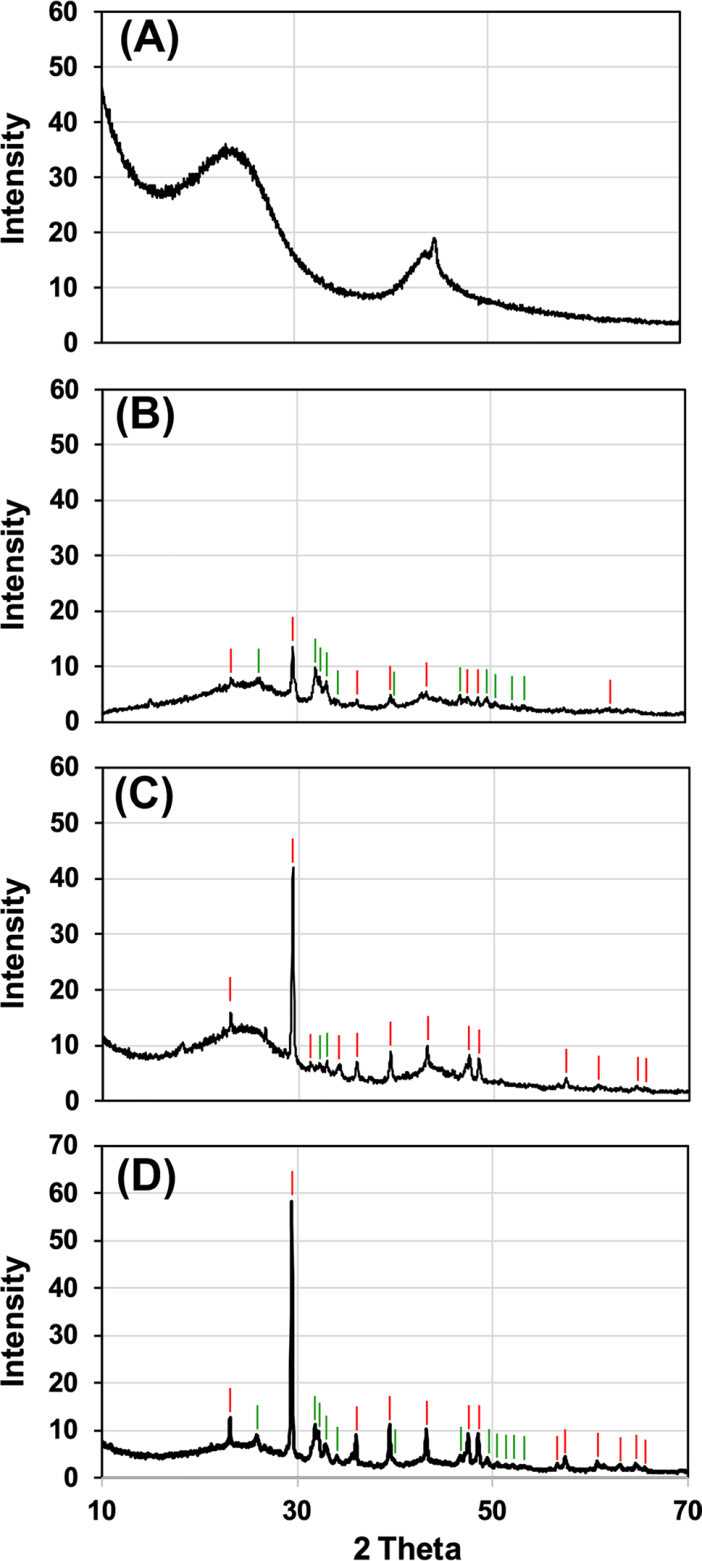
X-ray diffraction analysis of **A** activated carbon, **B** untreated biochar B9-B11, **C** B9-B11 after washing with BioDea (B9-B11^(BD)^), and **D** successive thermal activation (B9-B11^(BD−TA)^). Calcite and hydroxyapatite phases are labelled with red and green tags, respectively

**Fig. 6:**
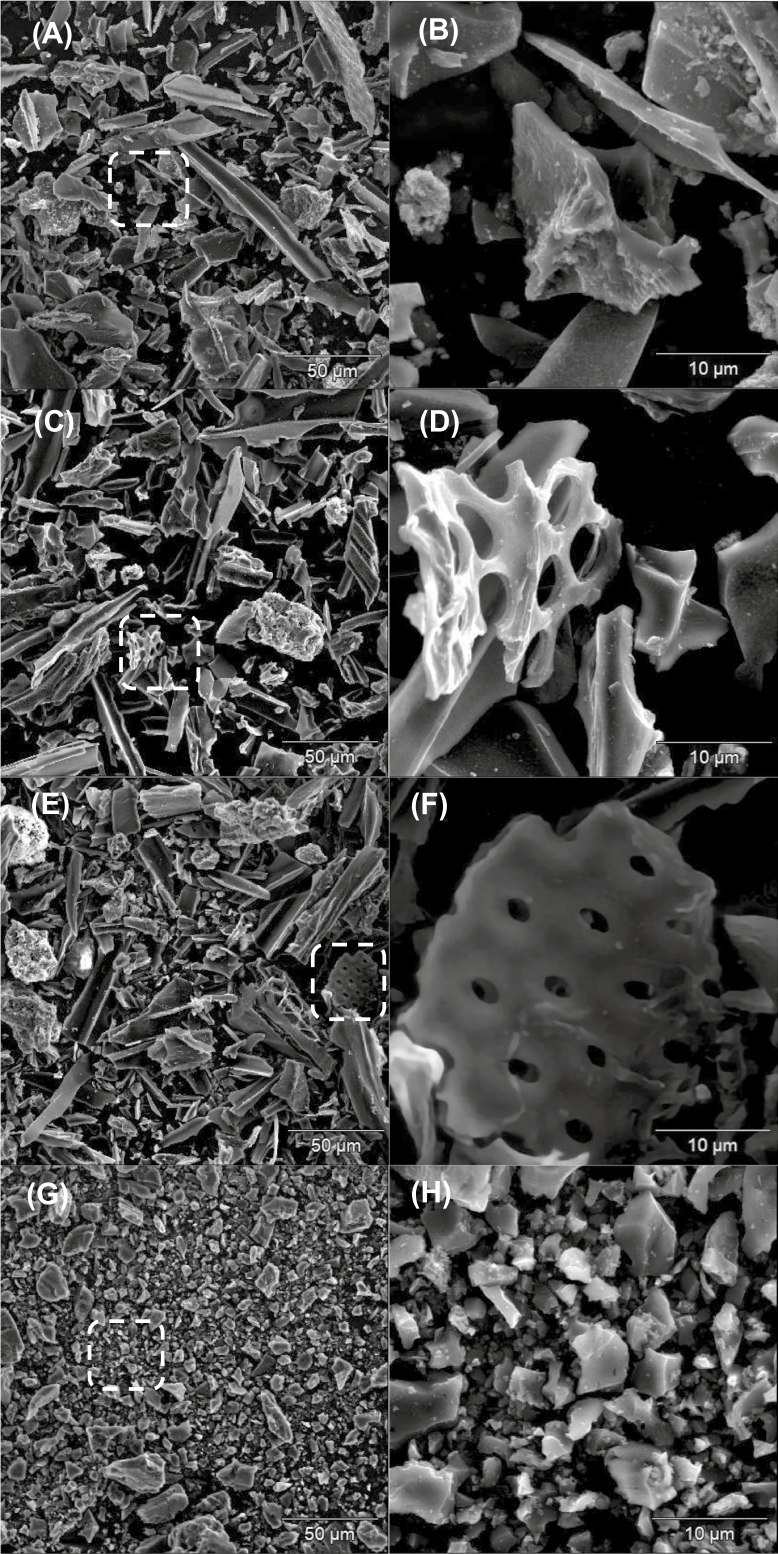
SEM images of B9-B11 (**A**, **B**), B9-B11^(BD)^ (**C**, **D**), B9-B11^(BD−TA)^ (**E**, **F**), and AC (**G**, **H**) taken at different magnifications. Dotted boxes identify the regions that underwent magnification

B9-B11 was selected for further characterization due to its higher SSA values after thermal activation (Fig. 3). These characterizations included an in-depth physisorption analysis for investigating microporosity/mesoporosity of the materials and pH_PZC_ (Table S12 of the *Supplementary material*), as well as thermogravimetric analysis (TGA, Fig. 4), X-ray diffraction (XRD, Fig. 5A–D), and scanning electron microscopy (SEM, Fig. 6A–H), which were performed on (i) the original material, as well as (ii) the one washed with BioDea (B9-B11^(BD)^) and (iii) the one washed and thermally activated (B9-B11^(BD−TA)^).

The sum of the t-plot microporous and BJH mesoporous areas was only slightly lower than the BET total SSA, thus suggesting a low contribution of macropores to the total surface area (Table S12). Although the three materials were characterized by a prevalent microporosity, the treatments performed on the biochar influenced the pore size distribution. In fact, the washing with BioDea strongly increased the microporosity, while decreasing mesoporosity. This result may be due to the ability of the washing solution to remove small size compounds that filled micropores, while higher size organic compounds present in the BioDea (e.g. polyphenolic compounds) could partially saturate the mesopores. Thermal activation showed an opposite effect, since a decrease in microporosity and an increase in mesoporosity were observed, consistently with a swelling-like temperature-dependent phenomenon and the thermal degradation of the pore-filling organic compounds.

The TGA highlighted an excellent resistance of both B9-B11 and B9-B11^(BD)^. In fact, the chars showed a very small weight loss (i.e. about 7%) in the investigated temperature range (i.e. up to about 500 °C). The thermal activation significantly increased this resistance, leading to a material behaving very similar to the AC in the investigated temperature range, being their weight losses approximately of 4.5% and 3.5%, respectively. It should also be noted that these results support the reliability of the surface area data, which were obtained after degassing at 200 °C.

XRD pattern for the AC (Fig. 5A) showed broad asymmetric peaks corresponding to 2θ≈25° and 45°, typically found in commercially available virgin activated carbons from vegetal feedstocks (Cheng et al. 2020; Wu et al. 2017) and attributable to the presence of amorphous carbon partially composed of sheets of randomly oriented aromatic carbon (Suganuma et al. 2008). In contrast, diffractograms of untreated biochar (Fig. 5B), after washing (Fig. 5C), and subsequent thermal activation (Fig. 5D) show completely different profiles from that of activated carbon, in accordance with the completely different nature of the feedstocks used. In detail, the XRD spectra of the three biochars show narrow peaks of increasing intensity from untreated biochar to BioDea-washed biochar and finally to thermally activated biochar, highlighting the increasing formation of crystalline phases along the treatments. These crystalline phases mainly consist of calcite and hydroxyapatite, as demonstrated by the comparison with XRD reference libraries. Although the presence of aromatic moieties in this type of material is a constant (Fan et al. 2023), no clear evidence of the occurrence of graphite-like sheets has been observed.

**Fig. 7:**
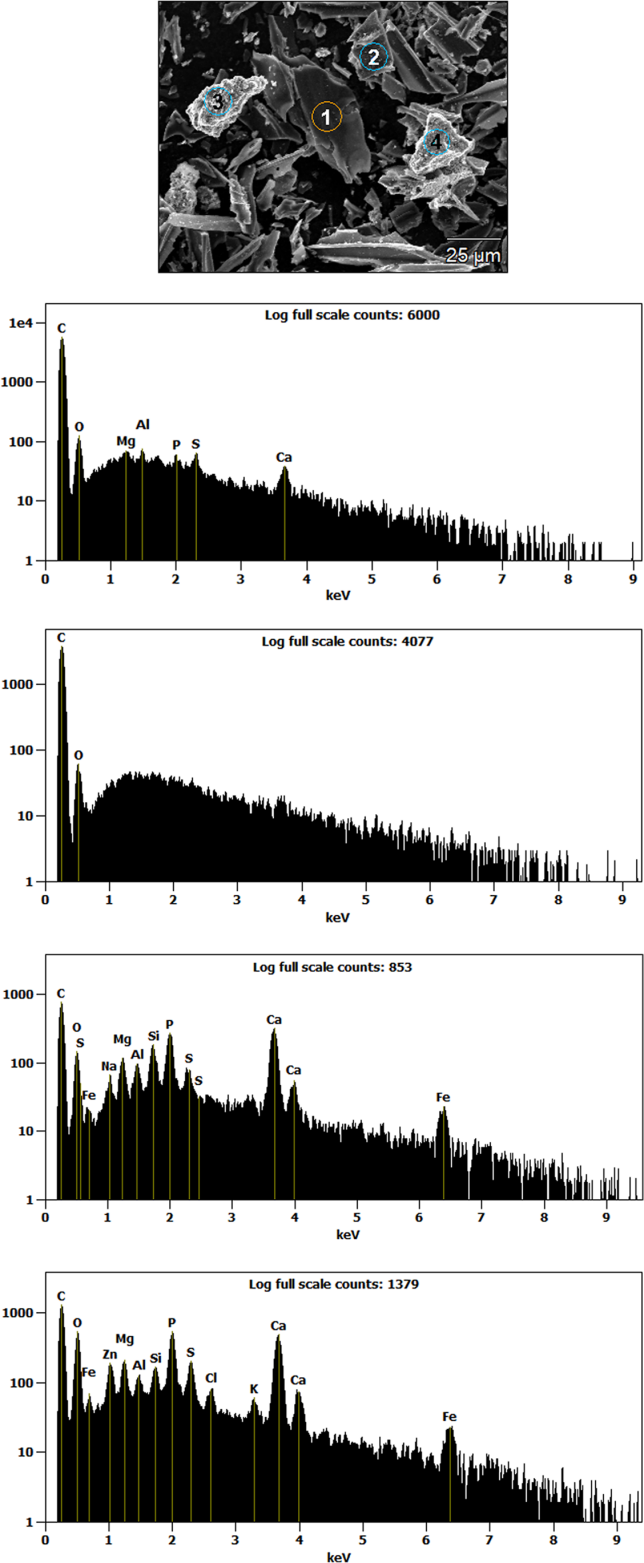
Representative SEM–EDX results obtained on the B9-B11^(^.^BD)^

SEM images (Fig. 6A–H) allowed making some considerations about the morphology of the investigated materials. As a general consideration, all biochars (Fig. 6A–F) exhibited a wider particle size distribution than AC (Fig. 6G–H). In fact, the latter consisted in particles dimensionally more homogeneous, most of them < 5 µm. This finding reflects the different characteristics of the feedstocks used for the preparation of the two kinds of materials. Particles of different shapes can be observed in all biochars. In particular, rather large particles with an elongated shape, smaller particles without a defined shape, and honeycomb-like shapes were highlighted, the latter after washing with BioDea. The SEM–EDX analysis allowed performing a semi-quantitative evaluation of the elemental composition of the particles; a representative example showing the results obtained for some data acquisitions performed on the B9-B11^(BD)^ is reported in Fig. 7. Most particles consisted almost completely in carbon (see panels 1–2), while others (see panels 3–4) contained also significant amounts of other elements such as P, S, Ca, Mg, Fe, and Al, thus highlighting the composite nature of the materials. This heterogeneous composition is in accordance with the heterogeneity of the mixed feedstocks and the recycled solution used for preparing and washing the material, respectively. More in detail, the presence of particles with a high Fe and Al content can be explained by the use of ferric chloride and aluminium polychloride during the sedimentation and clariflocculation stages of the wastewater treatment.

### Evaluation of adsorption capacity in ultrapure water

Sorption capacity of B9-B11^(BD)^ and B9-B11^(BD−TA)^ was evaluated towards MB and DY by kinetic and isotherm analyses in ultrapure water, using AC as reference material.

#### Kinetic test

As illustrated in Figure S8 of the *Supplementary Material*, appreciable removals were determined with B9-B11^(BD)^ for MB and especially with B9-B11^(BD−TA)^ for both dyeing agents, while no significant removal was observed for DY using the B9-B11^(BD)^ (data not reported), evidencing its poor adsorption capacity for this molecule. Considering that AC was used in kinetic tests in quantities an order of magnitude lower than biochar (see section S.6.1), its removal capacity was higher than that of biochar (Fig. S8). The data clearly showed that the highest and most stable uptake of dyeing agents was obtained with a contact time ranging from 60 to 120 min, while the increase to 180 min offered no improvement in the removal. These results highlighted a quite fast adsorption kinetic for all biochars that is encouraging for their use in real systems. According to the results of the adsorption kinetic studies, a contact time of 120 min was selected for the isotherm tests.

#### Adsorption isotherms

The interpretation of the experimental results of adsorption tests was attempted with both Langmuir and Freundlich models. However, the linearized Freundlich equation provided models with low statistical significance (low *R*^2^ values and *P* > 0.05, data not shown). Conversely, Langmuir equation generally produced significant models and was therefore used for data interpretation (see Table 2). Based on Langmuir *Q*_m_ data determined in ultrapure water, it was clear that the thermal activation significantly increased the sorption capacity of the biochar, leading for MB to a *Q*_m_ value about 8 times higher (62 vs. 7.6 mg g^−1^) and making detectable the maximum adsorption for DY (5.2 mg g^−1^). Based on the surface properties of the materials (i.e. SSA, porosity distribution, and surface charge), it is possible to propose mechanistic interpretations of the adsorption data obtained. The higher sorption of B9-B11^(BD−TA)^ is consistent with the higher SSA and mesoporosity of this material, both playing a relevant role in the adsorption of organic molecules and especially compounds with diameter larger than 2 nm, as in the case of DY. Interestingly, the thermally activated biochar provided *Q*_m_ values only 2.5 times lower than the one obtained with AC used here as a comparison (62 vs. 153 mg g^−1^ and 5.2 vs. 12 mg g^−1^ for MB and DY, respectively). These results agree with the higher SSA value found for AC (785 m^2^ g^−1^) compared to B9-B11^(BD−TA)^ (460 m^2^ g^−1^) (Table S12). MB exhibited higher *Q*_m_ values than DY both with B9-B11^(BD−TA)^ and AC, probably owing to the significantly different size of the two molecules (maximum diameter of about 1.4 and 2.6 nm for MB and DY, respectively), which determines a much lower surface area available for DY adsorption, rather than for MB removal. In any case, π-π interactions between aromatic moieties of the material and the dyes play a role in their adsorption. Conversely, our findings seem not to be related to a role of electrostatic attractions/repulsions between adsorbate and adsorbent. In fact, under the pH conditions of ultrapure water added with dyeing agents (pH = 5.9), B9-B11^(BD−TA)^ has a positive net surface charge and should therefore adsorb better DY than MB if the electrostatic sorption mechanism played an important role in governing dyes removal. A less important role of pure electrostatic interactions in the removal of dyeing agents by biochars was also observed by other researchers (Spagnoli et al. 2017; Sumalinog et al. 2018), who proposed different non-electrostatic interactions (i.e. dipole–dipole and π-π), and pore dimension as major driving forces for explaining sorption phenomena.

**Table 2:** Mean values (*n* = 3) and standard deviation (in bracket) of the Langmuir sorption maxima (*Q*_m_, mg g^−1^) determined for methylene blue (MB) and direct yellow 50 (DY) solutions in ultrapure water and wastewater (concentration range 25–100 mg L^−1^) in the presence of biochars (B9-B11^(BD)^ and B9-B11^(BD−TA)^, 4000 mg L^−1^) and commercially available activated carbon (AC, 400 mg L^−1^). Determination coefficients (*R*^2^) and *P*-values are also reported to highlight the linearity and statistical significance of the Langmuir models obtained with the experimental data

		Ultrapure water			Wastewater		
		*Q*_m_	*R*^2^	*P*-value	*Q*_m_	*R*^2^	*P*-value
MB	B9-B11^(BD)^	7.6 (0.2)	0.911–0.994	8.0E-7–8.2E-4	n.m	n.m	n.m
	B9-B11^(BD−TA)^	62 (4)	0.827–0.940	3.0E-4–4.5E-3	28 (4)	0.958–0.993	3.7E-3–2.1E-2
	AC	153 (1)	0.991–0.995	1.5E-4–3.9E-4	260 (3)	0.987–0.996	1.9E-3–6.7E-3
DY	B9-B11^(BD)^	n.m	n.m	n.m	n.m	n.m	n.m
	B9-B11^(BD−TA)^	5.2 (0.6)	0.920–0.975	1.6E-3–9.8E-3	13.6 (0.6)	0.997–0.999	6.5E-4–1.5E-3
	AC	12 (1)	0.989–0.998	4.7E-5–1.8E-4	21 (1)	0.970–0.982	8.9E-3–1.5E-2

*n.m.* not measurable

It is interesting to compare the best *Q*_m_ value determined here with those reported elsewhere for biochars obtained from mixtures of WWBs and BSs in proportions similar to that used for the preparation of B9-B11 (Table S11). Removal of MB has been previously investigated by some researchers (Chen et al. 2019; Cheng et al. 2013; Dai et al. 2022a, 2022b; Fan et al. 2016; Kenchannavar and Surenjan 2022; Xiang et al. 2023), while no previous study has been performed on DY. Our best result (62 mg g^−1^), obtained after washing and thermal activation of the B9-B11, is included in the wide range of literature *Q*_m_ data (8.1–115 mg g^−1^), but it ranks among the best literature values, being much higher than their mean and median (38 and 23 mg g^−1^).

### Evaluation of adsorption capacity in real wastewater

The results obtained for B9-B11^(BD−TA)^ in ultrapure water are encouraging to evaluate the potential of this material for the removal of dyes in real wastewater. Hence, adsorption isotherm and breakthrough column tests were conducted on the effluent from the clariflocculation of the same WWTP from which the BS was collected for biochar production.

#### Adsorption isotherms

Table 2 shows the results of modelling the experimental adsorption data in effluent wastewater, using the Langmuir equation. In this complex matrix, the removal of the two dyes by biochar and AC showed differences from what was observed in ultrapure water, thus highlighting the importance of evaluating the removal performances in real wastewater. However, the lower sorption efficiency of B9-B11^(BD)^ compared to B9-B11^(BD−TA)^ was confirmed, being *Q*_m_ values measurables only for the thermally activated material. Moreover, in agreement with findings in ultrapure water, AC showed also in real wastewater an adsorption capacity 2–9 times higher than the thermally activated biochar.

#### Breakthrough column tests

To evaluate the sorption capacity of B9-B11^(BD−TA)^ under experimental conditions closer to real applications of wastewater refining, breakthrough column tests were performed (see section S.6.1 for full details). A representative example of the trend of MB concentrations in the column effluent during the experiment is shown in Figure S9 of the *Supplementary Material*. A quantitative removal of MB was achieved for the first 8 L of filtered wastewater, which corresponds to the removal of about 413 mg of MB, i.e. about 11.8 mg/g of biochar. Then, MB effluent concentration starts to grow until it reaches the initial concentration of about 52 mg L^−1^ after the filtration of an additional 10 L of wastewater. Overall, the total removal of MB in the columns was about 21 mg/g of biochar, which is in very good agreement with the *Q*_m_ value determined by isotherm tests (28 mg g^−1^, Table 2).

## Conclusions

This study provided in-depth knowledge of the physicochemical properties of biochar produced by thermal conversion of sludge from predominantly industrial wastewater input, a topic poorly described in the literature, making interesting considerations about the biochar characteristics in relation to the production conditions, including type and percentage of sludge used as feedstock.

Post-synthesis chemical treatment of biochars was allowed for obtaining materials complying with EN 12915–1, even in the presence of sludge from the treatment of mixed industrial-municipal wastewater, with a percentage in the feedstock as high as 30%, thus achieving the “end-of-waste” condition for this sludge. In this regard, the use of a low-cost and sustainable “secondary product”, obtained within the gasification of waste vegetal biomass for energy production, should be considered a significant added value of the proposed process.

The subsequent thermal activation, carried out on biochar containing 30% of sludge, provided an increase in surface area up to 460 m^2^ g^−1^, without compromising the compliance with the aforementioned standard, thus allowing to obtain promising materials for the adsorption of organic micropollutants. This biochar was able to provide direct yellow 50 and methylene blue adsorption capacities, only 2–9 times lower than those achieved by a commercial activated carbon. These removals were obtained in real wastewater sampled from the WWTP from which the sludge used as feedstock derives, thus suggesting a possible closure of the “sludge cycle” inside the facility. As reported elsewhere (Castiglioni et al. 2021, 2022; Del Bubba et al. 2020), it should be stressed the importance of evaluating the removal capacity of non-conventional materials in comparison with reference ones (such as in this case activated carbons), since the reliability of the data obtained is enhanced.

### Supplementary Information

Below is the link to the electronic supplementary material.

Supplementary file1 (DOCX 883 KB)
