# Supplementary material for: Biochar from co-pyrolysis of biological sludge and woody waste followed by chemical and thermal activation: end-of-waste procedure for sludge management and biochar sorption efficiency for anionic and cationic dyes
Source: Environ Sci Pollut Res Int. 2024 May 9;31(24):35249–65. doi: 10.1007/s11356-024-33577-3 (PMC11136814; doi:10.1007/s11356-024-33577-3)
Supplement: Supplementary file 1 — Supplementary file1 (DOCX 883 KB) [file 11356_2024_33577_MOESM1_ESM.docx]

**Supplementary material of the Manuscript: “Biochar from co-pyrolysis of biological sludge and woody waste followed by chemical and thermal activation: end-of-waste procedure for sludge management and biochar sorption efficiency for anionic and cationic dyes”**

# Reagents, standards and materials

Hydrochloric acid, methylene blue tri-hydrate and dichloromethane were purchased from Carlo Erba Reagents (Cornaredo, Milano, Italy). Direct yellow 50 was supplied by Santa Cruz Biotechnology Inc. (Dallas, TX, USA). Sodium bicarbonate, calcium chloride, magnesium sulphate and isopropyl alcohol were obtained from Sigma-Aldrich (St. Louis, MO, USA). Sodium chloride (Fluka) was purchased from Honeywell Fluka™ (Charlotte, North Carolina, USA). Virgin vegetal activated carbon (AC) was purchased from SICAV (Chieti, Italy).

Concentrated nitric acid 70%, hydrogen peroxide 30% (trace metal analysis) and all standards (ICP-MS analysis grade) of elements investigated in the chars were supplied from Sigma-Aldrich. Ultrapure water (resistivity > 18 MΩ) was obtained from a Milli-Q system (Millipore, Billerica, MA, USA).

EPA 610 PAH mixture (Supelco, St. Louis, MO, USA), containing the following polycyclic aromatic hydrocarbons: naphthalene (N), acenaphthene (Acy), acenaphthylene (Ac), fluorene (Fl), anthracene (A), phenanthrene (P), fluoranthene (Flu), pyrene (Py), benzo(a)anthracene (BaA), chrysene (Ch), benzo(b)fluoranthene (BbFlu), benzo(k)fluoranthene (BkFlu), benzo(a)pyrene (BaPy), dibenzo(a,h)anthracene (BahA), indeno(1,2,3-cd)pyrene (Ipy) and benzo(ghi)perylene (BP), was employed. Naphthalene d-8 (N-d8), phenanthrene d-10 (P-d10), fluoranthene d-10 (Flu-d10), chrysene d-12 (Ch-d12), benzo(a)pyrene d-12 (BaPy-d12) and indeno(1,2,3-cd)pyrene d-12 (Ipy-d12) were purchased from Supelco.

The GC column employed for PAH evaluation was an SLB®-5ms fused silica capillary (10 m × 0.10 mm, 0.10 μm film thickness) from Supelco. The SPE cartridge used for the evaluation of polycyclic aromatic hydrocarbons contents in char was the cartridge Strata C18-E (500 mg, 6 mL, 55 μm, 70 Å) from Phenomenex (Torrance, CA, USA).

**Table S1** – Compound names and acronyms (in brackets), CAS, structure formulas and maximum absorption wavelengths of target analytes (λ, nm).

| **Compound** | **CAS** | **Structure formula** | **logK_ow_^a^** | **λ** |
| --- | --- | --- | --- | --- |
| Methylene Blue (MB) | 61-73-4 |  | 2.61 | 652 |
| Direct Yellow 50 (DY) | 3214-47-9 |  | -1.85 | 404 |

^a^ Calculated by Chemicalize online platform (ChemAxon, Budapest, HU)

# Characterization of biological sludges

The biological sludges used in this study were obtained from the Calice and Vernio wastewater treatment plants (WWTPs) operating in two areas of the textile industrial district of Prato (Italy). The characteristics of the sludges are shown in **Table S2**.

**Table S2** – Concentration of ash (% d.w.), total organic carbon (TOC, % d.w.), total nitrogen (N_TOT_, % d.w.), total phosphorus (P_TOT_, % d.w.), selected elements (mg kg^-1^ d.w.), polycyclic aromatic hydrocarbons (PAHs, mg kg^-1^ d.w.), total polychlorinated biphenyls (PCBs, mg kg^-1^ d.w.), sum of polychlorinated dibenzodioxins (PCDDs), polychlorinated dibenzofurans (PCDFs), and dioxin-like PCBs (ng kg^-1^ d.w., expressed on the basis of WHO TEQ), toluene (mg kg^-1^ d.w.), adsorbable organic halides (AOX, mg kg^-1^ d.w.), diethylhexyl phthalate (DEHP, mg kg^-1^ d.w.). Limits reported in the table refers to the Italian Legislative Decrees n. 99/1992 and 130/2018; n.a. = limit not available.

| **Parameter** | **Calice** | **Vernio** | **Limit** |
| --- | --- | --- | --- |
| pH | 8.5 | 7.5 | 5.5-11 |
| Ash | 23 | 47 | n.a. |
| TOC | 7.2 | 4.6 | ≥20 |
| N_TOT_ | 0.9 | 0.7 | >1.5 |
| P_TOT_ | 0.3 | 0.2 | <0.4 |
| As | <10 | <10 | <20 |
| Be | <2 | <2 | <2 |
| Cd | <10 | <10 | 20 |
| Cr | 142 | 163 | <200 |
| Cr (VI) | 3.8 | 2.7 | <2 |
| Hg | <1 | <1 | 10 |
| Ni | 205 | <10 | 300 |
| Pb | 17.6 | 12.5 | 750 |
| Cu | 40.3 | 89 | 1000 |
| Se | <10 | <10 | <10 |
| Zn | 130 | 1070 | 2500 |
| PAHs^(a)^ | 9.3 | 8.4 | <6 |
| PCBs | 0.62 | 0.48 | <0.8 |
| PCDD+PCDF+PCB Dioxin like | 12 | 10 | ≤25 |
| Toluene | <1 | <1 | ≤100 |
| AOX^(b)^ | <85 | <85 | <500 |
| Hydrocarbons (C10-C40)^(c)^ | 1890 | 135 | <1000 |

1. Sum of the following individual PAHs: Acenaphthene, Phenanthrene, Fluorene, Fluoranthene, Pyrene, Benzo[b]fluoranthene, Benzo[j]fluoranthene, Benzo[k]fluoranthene, Benzo[a]pyrene, Benzo[g,h,i]perylene, Indeno [1,2,3-c,d]pyrene, Dibenzo (a,h) anthracene, Benzo [a] anthracene, Chrysene, Benzo[e]pyrene, Dibenzo[a,e]pyrene, Dibenzo[a,l]pyrene, Dibenzo[a,i]pyrene, Dibenzo[a,h]pyrene.
2. Sum of Lindane, Endosulfan, Trichloroethylene, Tetrachloroethylene, Chlorobenzenes
3. Measured on the sample as such, without drying.

# Char characterization

## S.3.1 Elemental analysis

Aliquots of about 2 mg of finely grinded samples, dried at 120 °C for 24 hours, were analysed in triplicate using a FlashEA^®^ 1112 elemental analyser Thermo Fisher Scientific (Waltham, MA) equipped with a thermal conductivity detector. Data concerning C, H, N, S, and O analysis are presented in **Table S3**. The percentage content of oxygen was determined as follows: O(%) = 100 – (C% + H% + N% + S% + ash%). **Figure S1** illustrates the van Krevelen diagram of O/C vs H/C.

**Table S3** – Elemental composition of biochars and of pH of the zero-point charge (pH_PZC_) presented as mean (n = 3) and standard deviation (in bracket). Biochars marked with the same superscript letter are samples referring to different productions under the same experimental conditions.

| **Biochar** | **C (%)** | **H (%)** | **N (%)** | **S (%)** | **O (%)** | **pH_PZC_** |
| --- | --- | --- | --- | --- | --- | --- |
| B1 | 61.4 (0.4) | 2.1 (0.1) | 0.42 (0.04) | <0.05^(*)^ | 30 (1) | 5.8 (0.2) |
| B2 | 77.4 (0.2) | 1.2 (0.1) | 0.31 (0.02) | <0.05^(*)^ | 12 (2) | 9.6 (0.3) |
| B3^(a)^ | 79.8 (0.4) | 0.8 (0.3) | 0.18 (0.02) | <0.05^(*)^ | 12 (2) | 9.7 (0.3) |
| B4 | 55.2 (0.2) | 1.6 (0.1) | 2.1 (0.1) | <0.05^(*)^ | 30 (1) | 6.9 (0.2) |
| B5 | 64.0 (0.6) | 1.1 (0.1) | 0.94 (0.09) | <0.05^(*)^ | 11 (1) | 9.5 (0.5) |
| B6 | 57 (5) | 0.7 (0.4) | 0.6 (0.1) | <0.05^(*)^ | 15 (6) | 9.9 (0.3) |
| B7 | 47.5 (0.2) | 1.6 (0.1) | 2.0 (0.1) | <0.05^(*)^ | 20 (1) | 6.4 (0.3) |
| B8^(b)^ | 64.1 (0.2) | 1.2 (0.2) | 1.3 (0.1) | <0.05^(*)^ | 10 (2) | 9.5 (0.2) |
| B9^(c)^ | 64.9 (0.7) | 0.8 (0.3) | 0.9 (0.1) | <0.05^(*)^ | 6 (2) | 10.1 (0.3) |
| B10^(a)^ | 76.4 (0.2) | 0.45 (0.01) | 0.21 (0.02) | <0.05^(*)^ | 15 (1) | 9.6 (0.2) |
| B11^(c)^ | 63.3 (0.2) | 0.8 (0.5) | 0.9 (0.1) | <0.05^(*)^ | 8 (1) | 10.2 (0.3) |
| B12^(b)^ | 65.5 (0.4) | 1.4 (0.2) | 1.7 (0.1) | <0.05^(*)^ | 10 (1) | 9.6 (0.4) |

^(*)^ Detection limit

**Figure S1** – van Krevelen diagram for biochars produced at 450°C (B1 and B4), 650°C (B2, B5, B8 and B12), and 850°C (B3, B6, B9, B10, B11 and B12).

## S.3.2 Ash content

The ash content was determined by carefully weighing the material (about 4 g aliquots) up to constant weight (difference between two successive weighing < 0.5%), using equation (1),

$$\text{Ash }\left( \text{\%} \right)\text{=100×}\frac{\left( \text{M}_{\text{2}}\text{-}\text{M}_{\text{0}} \right)}{\left( \text{M}_{\text{1}}\text{-}\text{M}_{\text{0}} \right)}$$

where: M_0_ is the mass of the empty crucible after heating at 815°C for one hour; M_1_ is the mass of the crucible with a quantity of biochar equal to about 4 g dry weight (d.w.) accurately weighed; M_2_ is the mass of the crucible with the residual ash after combustion at 815°C for 4 hours. The ash percentage of the twelve biochars produced according to the experimental conditions reported in Table 1 of the main text are reported in **Table S4**.

## S.3.3 Physisorption analyses

The textural properties of the materials were determined by nitrogen adsorption and desorption experiments at -196°C using a Micromeritics Adsorption Analyzer (Norcross, GA, USA) model 3Flex. The specific surface area (SSA) was calculated by using the Brunauer-Emmet-Teller (BET) method in the relative pressure (P/P°) range of 0.01-1.0, selecting a linear portion of BET plot in compliance to the Rouquerol criteria (J. Rouquerol et al., In: Studies in Surface Science and Catalysis, edited by P. L. Llewellyn, F. Rodriquez-Reinoso, J. Rouqerol and N. Seaton, Elsevier 2007 Vol. 160). SSA values of the twelve biochars produced according to the experimental conditions reported in Table 1 of the main text are illustrated in **Table S4**. The total pore volume was calculated from the amount of nitrogen adsorbed at relative pressure (P/P°) nearest to 1.0. The mesopore size distribution was determined by the Barret-Joyner-Halenda (BJH) model applied to desorption data, while the assessment of microporosity was carried out by the t-plot model, using the Harkins-Jura model as thickness curve equation. For the porosimetry of chars, a minimum equilibrium interval of 10 s and 20 s were used respectively in the relative pressure (P/P°) ranges of 0.01-0.1 and 0.1-1.0, with a maximum relative tolerance of 5% of the targeted pressure and an absolute tolerance of 5 mmHg. Before analysis, chars were preliminarily degassed under nitrogen stream at 200°C for 1h, then evacuated under a high vacuum (<10^-2^ mbar), provided by an oil-based vacuum pump coupled with a high vacuum system, for 0.50h and finally activated in situ, by heating at 200 °C, at a rate of 5°C min^-1^, for 180 min.

## S.3.4 pH of the point of zero charge

The pH of the point of zero charge (pH_PZC_) of biochars and MAC was determined using the pH drift method using the following procedure. Stock solutions of 0.1 M NaCl were prepared at pH 2, 3, 4, 5, 6, 7, 8, 9, 10, and 11 (pH_i_) by adding 1 M NaOH or 1 M HCl. Aliquots of 50 mL of these solutions were then transferred to a series of volumetric flasks and 0.1 g of each sorbent was added. The flasks were sealed and the suspensions orbital shaken at 150 rpm for 32 h before the final pH value (pH_f_) of the supernatant was recorded. The difference between pH_i_ and pH_f_ (ΔpH) was plotted as a function of pH_i_, and the point of the intersection of the resulting curve with pHi axis was pH_PZC_. Values of pH_PZC_ of the twelve biochars produced according to the experimental conditions reported in Table 1 of the main text, are illustrated in **Table S3**, while the graphs obtained for pH_PZC_ determination are shown in **Figure S2**.

**Figure S2** – Point of zero charge of biochars produced by pyrolysis at 450°C (B1 and B4), 650°C (B2, B5, B8 and B12), and 850°C (B3, B6, B9, B10, B11 and B12).

**Table S4** – Ash content (%), concentrations of selected elements (µg L^-1^) and polycyclic aromatic hydrocarbons (PAHs, ng L^-1^), and values of specific surface area (SSA, m^2^ g^-1^) of biochars. Biochars marked with the same superscript letter are samples referring to different productions under the same experimental conditions. EN 12915-1 limits are also reported.

|  | **Ash** | **As** | **Cd** | **Cr** | **Hg** | **Ni** | **Pb** | **Sb** | **Se** | **PAHs*** | **SSA** |
| --- | --- | --- | --- | --- | --- | --- | --- | --- | --- | --- | --- |
| B1 | 6.7 | <1** | <0.1** | 7.7 | <0.1** | 1.9 | <0.5** | 0.27 | <0.1** | 165 | 34.3 |
| B2 | 8.7 | <1** | <0.1** | 33 | <0.1** | <0.5** | <0.5** | <0.1** | <0.1** | 110 | 336 |
| B3^(a)^ | 6.9 | 2.4 | <0.1** | 46 | <0.1** | <0.5** | <0.5** | 2.2 | 0.69 | 21 | 552 |
| B4 | 11.8 | 25 | <0.1** | 66 | 0.11 | 3.8 | <0.5** | 6.2 | 1.1 | 136 | 73.7 |
| B5 | 22.5 | <1** | <0.1** | 34 | <0.1** | <0.5** | <0.5** | 1.1 | 0.38 | 138 | 258 |
| B6 | 27.2 | <1** | <0.1** | 18 | <0.1** | <0.5** | <0.5** | 3.2 | 1.6 | 29 | 370 |
| B7 | 29.0 | 5.1 | <0.1** | 19 | <0.1** | 1.4 | <0.5** | 4.2 | 2.0 | 0.9 | 16.9 |
| B8^(b)^ | 23.3 | 4.5 | <0.1** | 59 | <0.1** | <0.5** | <0.5** | 2.7 | 0.43 | 5.4 | 270 |
| B9^(c)^ | 27.4 | <1** | <0.1** | 22 | 0.8 | <0.5** | <0.5** | 0.16 | <0.1** | 12 | 300 |
| B10^(a)^ | 7.3 | <1** | <0.1** | 8.8 | 0.4 | <0.5** | <0.5** | 0.11 | <0.1** | 23 | 580 |
| B11^(c)^ | 26.3 | 2.6 | <0.1** | 60 | <0.1** | <0.5** | <0.5** | 2.5 | 0.61 | 12 | 319 |
| B12^(b)^ | 21.3 | 3.5 | <0.1** | 42 | <0.1** | <0.5** | <0.5** | 2.7 | 0.37 | 5.3 | 285 |
| EN 12915-1 | 15 | 10 | 0.5 | 5 | 0.3 | 15 | 5 | 3 | 3 | 20 | n.r. |

* PAHs regulated by the EN 12915-1: fluoranthene, benzo(b)fluoranthene, benzo(k)fluoranthene, benzo(a)pyrene, indeno(1,2,3-c,d)pyrene, and benzo(g,h,i)perylene; ** Limits of quantification; n.r. = not reported.

## S.3.5 Thermogravimetric analysis

## Thermogravimetric analysis (TGA) was conducted under N_2_ atmosphere (100 mL/min) on a Seiko Instruments Inc. (Chiba, Japan) TGA analyser, model EXSTAR 6200. Heating ramp was from 40 to 450°C, at 10°C/min. All samples were stabilized to constant weight at 40 °C before running each measurement.

## S.3.6 X-ray diffraction analysis

X-ray diffraction (XRD) was adopted to investigate the occurrence of amorphous and/or graphitic carbon as well as other crystalline phases in the selected biochars and in the commercial activated carbon. XRD was performed by using the Bruker (Billerica, MA, USA) New D8 Da Vinci X-ray diffractometer (radiation Cu-Kα1 = 1.54056Å, 40 kV x 40 mA) equipped with a Bruker LYNXEYE-XE detector. The scans were performed between 5 and 70° in 2theta, with increments of 0.034° and a time/step of 0.5 s on a flat sample holder. Data were analysed with the software EVA (version 6.0, Bruker AXS) and the international database PDF4+2021.

S.3.7 Scanning electron microscopy-Energy dispersive X-ray spectroscopy

A strip of carbon tape (conductive double-sided tape) was fixed on an aluminium support and a spatula tip of the material, previously grinded and sieved at 45 µm, was deposited on the strip. The excess powder was removed with a jet of nitrogen. The samples were analysed at 10 kV and at three different magnifications, i.e., x80 (250 µm scale), x500 (50 µm scale) and x3000 (10 µm scale) using a Hitachi (Tokyo, Japan) SU3800 SEM equipped with a Silicon Drift EDS Detector, model Ultim Max 40 (Oxford Instruments NanoAnalysis, High Wycombe, United Kingdom).

## S.3.8 Water-extractable substances

According to the EN 12902:2004 standard method, 10 grams of char are put in contact with 1 L of extraction water and the solution is shaken for 24 hours at 25°C. Extraction water was a NaHCO_3_ 0.5 mM, CaCl_2_ 0.3 mM and MgSO_4_ 0.2 mM aqueous solution (pH=7.5±0.2). Afterwards, the suspension obtained was filtered on a 0.4 µm polycarbonate membrane (Sigma-Aldrich, St. Louis, MO, USA) and divided in two aliquots for metal and PAH analysis.

## S.3.8.1 Analysis of water extractable metals

The filtered water sample (100 mL aliquots) was treated with 1 mL of concentrated nitric acid, spiked with 1% Au 1000 mg L^-1^ solution and directly analysed by an ICP-MS model 7700X (Agilent Technologies, Santa Clara, CA, U.S.A.) under the following experimental conditions: (i) plasma RF power 1450W, (ii) plasma gas flow (Ar) 15 L min^-1^ and (iii) auxiliary gas flow (Ar) 1.03 L min^-1^. Total analysis time, including washing procedure of the system, was about 5 minutes. The entire procedure is programmed and automatically controlled by the MassHunter Workstation software for ICP-MS G7201B ver. B01.02 Build 349.8 patch 2 (Agilent Technologies).

## S.3.8.2 Analysis of water extractable PAHs

PAHs were analysed according to the following internal method: 2.0 L of filtered solution were spiked with 5 mL of methanol, stirred and then loaded on a C18-SPE cartridge previously conditioned with (i) 4 mL of isopropyl alcohol, (ii) 4 mL of a 15/85 (*v/v*) isopropyl alcohol/water mixture and (iii) 25 mL of methanol. Afterward, the cartridge was dried under vacuum and the elution of the selected compounds was carried out with 6 mL of dichloromethane. The recovered solution was concentrated under a gentle nitrogen stream to 50 µL and analysed by a Shimadzu (Kyoto, Japan) gas chromatograph model GC2010 equipped with an AOC-20i auto-injector (Shimadzu) and an AOC-20s auto-sampler (Shimadzu) and coupled with a QP2010 Plus mass spectrometer (Shimadzu). The GC column employed was an SLB®-5ms fused silica capillary (10 m × 0.10 mm, 0.10 μm particle size) from Supelco.

The GC analysis was performed according to the following conditions: (i) 1 μL of sample was injected in splitless mode, (ii) injector temperature: 320°C, (iii) column flow: 1.2 mL min^-1^ and (iv) constant linear velocity: 69.2 cm s^-1^.

Temperature programme: 60 °C initial temperature for 2 min, from 60°C to 160 °C in 2.2 min, from 160°C to 200°C in 2.0 min, from 200°C to 210°C in 3.3 min, from 210°C to 225° C in 15.0 min. from 255°C to 244°C in 6.3 min, from 240°C to 320°C in 1.9 min, final isotherm for 1.0 min. MS conditions: ion source temperature: 230°C; interface temperature: 280°C; detector voltage: 0.5 kV; analysis mode: single ion monitoring (SIM), see **Table S5** for the complete list of the selected ions. Instrumental control and data processing were carried out by the GCMS Solution software, version 2.50 (Shimadzu).

The figure of merits of the instrumental method – i.e. limits of detection (IDLs), limits of quantification (IQLs), linearity and precision – obtained by replicated injections of standard solutions in dichloromethane, are shown in **Table S6**. IDLs and IQLs were taken as the minimum concentrations of target analytes that give rise to a signal to noise ratio (S/N) equal to 3 and 10, respectively. The linearity was investigated by replicated analysis (n=5) of standard solutions from five calibration levels of PAHs. For each analyte concentration ranges were chosen starting from the IQLs up to three magnitude orders expressed as ng injected. Intra-day (RSD%_intra_) and inter-day (RSD%_inter_) precision were evaluated by ten replicated injections of standard solutions, at concentration levels twice higher than IQLs.

**Table S5** – GC-MS retention time (Rt), quantifier and qualifier ions of the investigated PAHs and selected labelled surrogate standards. Meaning of target analyte acronyms is reported in section S.1.

| **Compound** | **Rt [min]** | **Quantifier ion** | **Qualifier ion** |
| --- | --- | --- | --- |
| N | 3.5 | 128 | 127 |
| Acy | 4.4 | 152 | 151 |
| Ac | 4.5 | 153 | 154 |
| Fl | 4.9 | 165 | 165 |
| P | 5.7 | 178 | 176 |
| A | 5.8 | 178 | 176 |
| Flu | 7.0 | 202 | 200 |
| Py | 7.4 | 202 | 200 |
| BaA | 10.5 | 228 | 226 |
| Ch | 10.6 | 228 | 226 |
| BbFlu | 15.8 | 252 | 250 |
| BkFlu | 16.1 | 252 | 250 |
| BaPy | 18.0 | 252 | 250 |
| Ipy | 27.3 | 276 | 274 |
| BahA | 28.4 | 278 | 276 |
| BP | 29.8 | 276 | 274 |
| N-d8 | 3.5 | 136 | 134 |
| P-d10 | 5.7 | 188 | 184 |
| Flu-d10 | 7.0 | 212 | 208 |
| Ch-d12 | 10.5 | 240 | 236 |
| BaPy-d12 | 18.0 | 264 | 260 |
| Ipy-d12 | 27.3 | 288 | 284 |

In order to evaluate the SPE apparent recovery, three 2 L aliquots of extraction water were fortified with labelled compounds (5 ng L^-1^ for N-d8, P-d10, Flu-d10 and Ch-d12 and 10 ng L^-1^ for BaP-d12 and Ipy-d12). The spiked samples were subjected to the SPE procedure, followed by the GC-MS analysis; the resulting mean areas (n=3) were compared to the mean areas (n=3) obtained by spiking 50 µL of dichloromethane with the same amounts of mass labelled compounds.

**Table S6 –** Instrumental figure of merits of GC-MS analysis of PAHs. The meaning of target analyte acronyms is reported in section S.1.

| **Compound** | **IDL**  **(ng injected)** | **Linearity range**  **(ng injected)^a^** | **R^2^** | **RSD%_intra_** | **RSD%_inter_** |
| --- | --- | --- | --- | --- | --- |
| N | 0.001 | 0.005 – 52.64 | 0.9996 | 2.1 | 2.8 |
| Acy | 0.001 | 0.004 – 52.98 | 0.9998 | 3.3 | 3.5 |
| Ac | 0.001 | 0.004 – 52.98 | 0.9994 | 2.4 | 2.8 |
| Fl | 0.001 | 0.005 – 5.40 | 0.9975 | 3.1 | 3.5 |
| P | 0.002 | 0.007 – 5.34 | 0.9996 | 3.5 | 3.8 |
| A | 0.002 | 0.007 – 5.37 | 0.9994 | 3.7 | 4.0 |
| Flu | 0.001 | 0.005 – 10.20 | 0.9994 | 3.2 | 3.3 |
| Py | 0.001 | 0.005 – 4.97 | 0.9987 | 3.4 | 3.5 |
| BaA | 0.003 | 0.01 – 26.63 | 0.9997 | 4.4 | 4.5 |
| Ch | 0.005 | 0.02 – 26.63 | 0.9994 | 4.2 | 4.4 |
| BbFlu | 0.004 | 0.015 – 10.87 | 0.9977 | 5.3 | 5.5 |
| BkFlu | 0.004 | 0.015 – 5.39 | 0.9983 | 5.1 | 5.4 |
| BaPy | 0.005 | 0.02 – 27.9 | 0.9995 | 4.8 | 5.2 |
| BahA | 0.008 | 0.03 – 29.16 | 0.9994 | 5.2 | 5.3 |
| Ipy | 0.006 | 0.025 – 26.75 | 0.9986 | 5.4 | 5.5 |
| BP | 0.008 | 0.030 – 3.15 | 0.9948 | 5.5 | 5.6 |
| N-d8 | 0.001 | 0.005 – 51.37 | 0.9998 | 1.9 | 2.2 |
| P-d10 | 0.002 | 0.007 – 5.52 | 0.9997 | 2.7 | 3.1 |
| Flu-d10 | 0.001 | 0.005 – 12.33 | 0.9995 | 2.8 | 3.0 |
| Ch-d12 | 0.005 | 0.02 – 24.53 | 0.9997 | 3.2 | 3.5 |
| BaPy-d12 | 0.005 | 0.02 – 26.8 | 0.9993 | 4.2 | 4.8 |
| Ipy-d12 | 0.006 | 0.02 – 21.15 | 0.9987 | 5.1 | 5.3 |

^a^ The lower limits of the linearity ranges represent IQLs.

The following deuterated PAHs were used for calculating apparent recoveries: (i) N-d8 for N, (ii) P-d10or 3-ring PAHs, (iii) Flu-d10 for Flu, (iv) Ch-d12 for 4-ring PAHs, (v) BaP-d12 for 5-ring PAHs and (vi) Ipy-d12 for 6-ring PAHs. The recoveries of mass labelled analytes are detailed in **Figure S3**, and the detection (MDLs) and quantification (MQLs) limits of the whole analytical procedure are shown in **Table S7**. The overall MQLs in extraction water were found in the range of 0.13 – 0.9 ng/L.


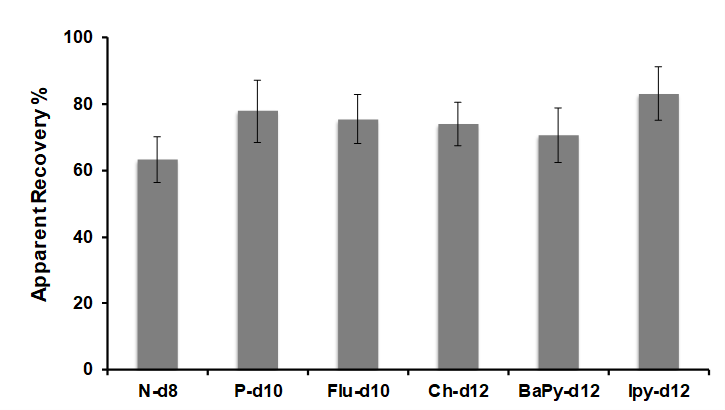


**Figure S3** – Mean percentage values of apparent recovery for the whole analytical procedure of PAH analysis. The meaning of target analyte acronyms is reported in section S.1.

**Table S7 –** Method detection (MDL) and quantification (MQL) limits (n=5) for the whole SPE-GC-MS procedure of PAH analysis. Values in bracket represent the standard deviation. The meaning of target analyte acronyms is reported in section S.1.

| **Compound** | **MDL (ng L^-1^)** | **MQL (ng L^-1^)** |
| --- | --- | --- |
| N | 0.050 (0.001) | 0.20 (0.01) |
| Acy | 0.032 (0.001) | 0.13 (0.01) |
| Ac | 0.032 (0.001) | 0.13 (0.01) |
| Fl | 0.040 (0.001) | 0.16 (0.01) |
| P | 0.056 (0.002) | 0.22 (0.01) |
| A | 0.056 (0.002) | 0.22 (0.01) |
| Flu | 0.042 (0.001) | 0.17 (0.01) |
| Py | 0.043 (0.001) | 0.17 (0.01) |
| BaA | 0.086 (0.004) | 0.34 (0.02) |
| Ch | 0.171 (0.008) | 0.68 (0.03) |
| BbFlu | 0.134 (0.007) | 0.54 (0.03) |
| BkFlu | 0.134 (0.007) | 0.54(0.03) |
| BaPy | 0.179 (0.009) | 0.71 (0.04) |
| BahA | 0.226 (0.012) | 0.90 (0.05) |
| Ipy | 0.188 (0.010) | 0.75 (0.04) |
| BP | 0.226 (0.013) | 0.90 (0.05) |

The concentrations of the selected PAHs in the twelve biochars produced according to the experimental conditions reported in Table 1 of the main text are illustrated in **Table S4**.

# Characterization of the wastewater used for adsorption studies

Effluent wastewater from the clariflocculation stage of the Calice WWTP, operating in the industrial textile district of Prato (Italy), was used for assessing the sorption capacity of the best performing biochar in tests with ultrapure water. This WWTP basically consisted in equalization, primary sedimentation, biological oxidation, secondary sedimentation, clariflocculation (i.e., the treatment stage after which wastewater was collected), and a final polishing treatment with ozone. **Table S8** illustrates the characterization of the effluent wastewater for a number of parameters routinely analysed.

**Table S8** – Characteristics of the effluent wastewater from the clariflocculation stage of the Calice WWTP.

| Parameter | Value |
| --- | --- |
| pH | 7.83 |
| SST (mg/L) | 9.1 |
| COD (mg/L) | 68 |
| BOD (mg/L) | 17 |
| N_TOT_ (mg/L) | 6.0 |
| N-NH_4_^+^ (mg/L) | 1.5 |
| N-NO_3_^-^ (mg/L) | 3.8 |
| N-NO_2_^-^ (mg/L) | 0.10 |
| P_TOT_ (mg/L) | 1.09 |
| MBAS (mg/L) | 0.38 |
| BiAS (mg/L) | 0.25 |
| Total surfactants (mg/L) | 0.64 |

# Optimization of the washing protocol of biochars with BioDea^®^

The chemical activation of the biochar by washing with the BioDea^®^ solution was performed after optimization of the mixing ratio and contact time. In the optimized conditions the protocol consisted in the stirring of a 1/10 (w/w) biochar/BioDea^®^ mixture for 30 minutes. **Table S9** illustrates the characterization of the BioDea^®^ solution.

**Table S9** – Characteristics of the BioDea^®^ solution used in this study for the chemical activation of biochars, presented as mean (n = 3) and standard deviation (in bracket).

|  | **Unit** | **Value** |
| --- | --- | --- |
| pH | - | 4.0 (0.5) |
| Acetic acid | % v/v | 2.2 (0.2) |
| Phenols | gr kg^-1^ | 2.96 (0.08) |
| Polyphenols | gr kg^-1^ | 24 (2) |
| As | µg L^-1^ | 1.16 (0.07) |
| Cd | µg L^-1^ | 5.5 (0.4) |
| Cr | µg L^-1^ | 1.64 (0.05) |
| Hg | µg L^-1^ | 0.29 (0.03) |
| Ni | µg L^-1^ | 14.9 (0.7) |
| Pb | µg L^-1^ | 61 (3) |
| Sb | µg L^-1^ | 3.1 (0.2) |
| Se | µg L^-1^ | 7.3 (0.8) |

# Kinetic and isotherm tests

The evaluation of biochar and activated carbon adsorption performance towards MB and DY was carried out by kinetic and isotherm tests, performed in triplicate. The kinetic and isotherm adsorption tests were performed by maintaining in agitation aqueous solutions containing dyeing agents and biochar under the following experimental conditions: (i) magnet agitator speed 500 rpm; (ii) absence of light; (iii) room temperature (20±2°C). The suspensions were centrifuged at 30000 x *g*, for 10 minutes, at 10°C (MPW-351R, MPW Med. Instruments, Warsaw, Poland) and supernatant aliquots were collected and passed through Sartorius Minisart SRP25 syringe filters (porosity 0.45 µm) before UV-Vis analysis. A control sample without the sorbent was run in parallel.

Stock solutions of MB and DY, both at concentration of 1000 mg/L, were prepared and diluted to obtain concentrations of 25, 50, 75 and 100 mg/L. Ultrapure water and the effluent wastewater from the secondary sedimentation of the Calice WWTP were used for preparing the solutions of dyeing agents, depending on the kind of test performed. An UV–Vis spectrophotometer HACH DR/4000 U was used to determine MB and DY concentrations before and after adsorption, monitoring absorbance at their maximum absorption visible wavelengths, i.e. 652 nm and 404 nm, respectively. Sample solutions with absorbance values out of the calibration range were properly diluted.

S.6.1 Adsorption kinetics

Adsorption kinetics on biochars and AC were investigated separately for MB and DY by maintaining in rotation at T = 20 °C test bottles containing 100 mg/L of the target analyte and 4000 mg/L of biochars or 400 mg/L of AC. Contact times of 5, 10, 20, 30, 60, and 180 minutes were investigated for both analytes. Additional contact times of 300 and 1440 minutes were used only for DY, in order to clarify desorption phenomena of this dying agent. The suspensions were finally centrifuged at about 1400xg for 6 minutes (MPW-351R, MPW Med. Instruments, Warsaw, Poland). Supernatant aliquots were collected and passed through Sartorius Minisart SRP25 syringe filters (porosity 0.45 µm) before UV-Vis analysis.

S.6.2 Adsorption isotherms

Langmuir and Freundlich equations were used to fit the adsorption isotherms data, as described in section S.6.2 of the *Supplementary material*. More specifically, the following Langmuir adsorption-isotherm equation was adopted:

$Q_{e}=\frac{Q_{m}\cdot k\cdot C_{e}}{1+(k\cdot C_{e})}$ (1)

where $C_{e}$ is the equilibrium adsorbate concentration (mg L^-1^), $Q_{e}$ the mass of adsorbate per mass unit of adsorbent at equilibrium (mg g^-1^), $Q_{m}$ the maximum mass adsorbed at equilibrium conditions per mass unit of adsorbent (mg g^-1^) and $k$ (L mg^-1^) the empirical constant with units of inverse of concentration $C_{e}$. The linear form of the above-mentioned equation is reported in the equation 2,

$\frac{C_{e}}{Q_{e}}=\frac{1}{Q_{m}\cdot k}+\frac{1}{Q_{m}}\cdot C_{e}$ (2)

which represents a straight line with slope $Q_{m}$^-1^ and intercept ($Q_{m} k$)^-1^ .

Accordingly, linearized Langmuir adsorption-isotherm equation allows for estimating both the $Q_{m}$ and $k$, the latter representing the inverse of the equilibrium concentration of the adsorbate at half saturation. Accordingly, *k* represents a measure of the affinity of the analyte for the sorbent.

The Freundlich isotherm model can be written as:

$\log Q_{e}=\log K_{F}+\frac{1}{n}\cdot\log C_{e}$ (3)

where $Q_{e}$ and $C_{e}$ have the same aforementioned meaning, while $K_{F}$ and $n$ are the Freundlich constants related to adsorption capacity and intensity, respectively.

Isotherm adsorption experiments were conducted in triplicate using seven concentration levels (i.e. 25, 35, 50, 60, 75, 90, and 100 mg/L) of MB and five different concentration levels (30, 40, 50, 60, and 70 mg/L) of DY, keeping constant the concentration of the sorbent material (i.e. 100 mg/L for biochars and 10 mg/L for AC). These experiments were carried out by maintaining the test bottles in rotation for 1 h at T = 20 °C, in accordance with the results of the kinetic tests.

S.6.3 Breakthrough column tests

Breakthrough column tests were carried out by passing fortified wastewater through columns filled with about 35 g of biochar (sieved at 45 µm). Effluent wastewater from the Calice WWTP was fortified with MB at a concentration of about 50 mg L^-1^ and passed through the column at a flow rate of approximately 180 mL h^-1^. In detail, the device used includes an array of 5 columns and 5 in-line filter units (0.45 μm). Each column is fed with the wastewater to be treated by a multi-channel peristaltic pump. Each column has an internal diameter of 21 mm and a total height of 15 cm. Glass wool is placed on the surface of the bed to avoid its disturbance during the continuous introduction of the wastewater.

# Results of the multivariate PLS models

The significance of the PLS models was evaluated based on Fitting (R^2^) and prediction (Q^2^) determination coefficients reported in **Table S10**, considering the value of 0.5 as the statistically significance threshold.

**Table S10** – Fitting (R^2^) and prediction (Q^2^) ability of the PLS regression model towards selected product and environmental characteristics of biochars, based on multilevel (i.e. pyrolysis temperature, contact time, sludge percentage) and qualitative (feedstock and sludge type) factors. Bold values refer to R^2^ and Q^2^ ≥ 0.5.

| Fitted/Predicted Variable | R^2^ | Q^2^ |
| --- | --- | --- |
| Ash content | **0.98** | **0.92** |
| PAHs release | **0.61** | **0.50** |
| As release | **0.78** | **0.51** |
| Cr release | 0.43 | 0.35 |
| Hg release | **0.56** | 0.30 |
| Ni release | **0.69** | **0.65** |
| Pb release | **0.72** | 0.45 |
| Sb release | **0.90** | **0.60** |
| Se release | **0.81** | **0.70** |
| Surface area | **0.91** | **0.80** |

**Figures S4-S7** illustrate the contour plots, built through the combination of the categorical variables “wood waste biomass” and “sludge type” and fixing CT at 90 min, describing the trend of parameters included in the EN 12915-1 as a function of pyrolysis temperature and sludge percentage.

**Figure S4** – Contour plots describing the trends of (A) surface area, (B) ash, (C) PAH release, (D) As release, (E) Ni release, (F) Sb release, and (G) Se release as a function of pyrolysis temperature and sludge percentage (contact time: 90 min; wood waste: oak; sludge: Calice WWTP).

**Figure S5** – Contour plots describing the trends of (A) surface area, (B) ash, (C) PAH release, (D) As release, (E) Ni release, (F) Sb release, and (G) Se release as a function of pyrolysis temperature and sludge percentage (contact time: 90 min; wood waste: oak; sludge: Vernio WWTP).

**Figure S6** – Contour plots describing the trends of (A) surface area, (B) ash, (C) PAH release, (D) As release, (E) Ni release, (F) Sb release, and (G) Se release as a function of pyrolysis temperature and sludge percentage (contact time: 90 min; wood waste: poplar; sludge: Calice WWTP).

**Figure S7** – Contour plots describing the trends of (A) surface area, (B) ash, (C) PAH release, (D) As release, (E) Ni release, (F) Sb release, and (G) Se release as a function of pyrolysis temperature and sludge percentage (contact time: 90 min; wood waste: poplar; sludge: Vernio WWTP).

# Characterization and sorption capacity of the best performing materials in comparison with literature

**Table S11** illustrates the values of BET total surface area and maximum adsorption capacity towards methylene blue (Q_m_, mg g^-1^) of biochars produced in this study (B9-B11^(BD)^ = washed with BioDea and B9-B11^(BD-TA)^ = washed with BioDea and thermally activated) in comparison with the corresponding data reported in literature for biochars produced using mixtures of vegetal biomass and biological sludge as feedstock.

In order to investigate the effect of the chemical and thermal treatments on parameters possibly linked to the sorption efficiency of biochars, the porosity distribution of the materials, together with pH_PZC_ were determined on B9-B11, B9-B11^(BD)^, and B9-B11^(BD-TA)^ (**Table S12**).

**Table S11** – BET (m^2^ g^-1^) total surface area and Q_m_ (mg g^-1^) for methylene blue in ultrapure water of B9-B11^(BD)^, B9-B11^(BD-TA)^, and biochars produced elsewhere from mixtures of biological sludge (BS) and vegetal biomass (VB). BS/VB = blending ratio.

| **Feedstock** | **Treatment** | **BS/VB** | **BET** | **Q_m_** | **Reference** |
| --- | --- | --- | --- | --- | --- |
| BS and oak sawdust (B9-B11^(BD)^) | Post-pyrolysis BioDea^®^ washing | 30/70 | 389 | 7.6 | This study |
| BS and oak sawdust (B9-B11^(BD-TA)^) | Post-pyrolysis BioDea^®^ washing + thermal activation | 30/70 | 460 | 62 | This study |
| BS and pine sawdust | - | 50/50 | 168 | 13 | [1] |
| BS and rice husk | - | 50/50 | 29 | 23 | [2] |
| BS and tea waste | - | 50/50 | n.i. | 13 | [3] |
| BS and sodium lignosulfonate | - | 20/80 | 243 | 115 | [4] |
| BS and lignin | - | 50/50 | 34 | 38 | [5] |
| BS and corn straw | - | 30/70 | n.i. | 8.1 | [6] |
| BS and corn straw | Pre-pyrolysis KOH impregnation | 30/70 | n.i. | 83 | [6] |
| BS and corn straw | Post-pyrolysis KOH impregnation | 30/70 | n.i. | 10 | [6] |
| BS and corn straw | Pre-pyrolysis H_3_PO_4_ impregnation | 30/70 | n.i. | 75 | [6] |
| BS and corn straw | Post-pyrolysis H_3_PO_4_ impregnation | 30/70 | n.i. | 8.4 | [6] |
| BS and coconut shell | - | 50/50 | n.i. | 32 | [7] |

n.i. = not investigated; n.a. = not available; [1] G. Cheng et al. Desalination and Water Treatment 51 (2013) 7081–7087; [2] S. Chen et al. Journal of Molecular Liquids 285 (2019) 62–74; [3] S. Fan et al. Journal of Molecular Liquids 220 (2016) 432–441; [4] Q. Dai et al. Journal of Analytical and Applied Pyrolysis 165 (2022) 105586; [5] Q. Dai et al. Fuel 324 (2022) 124587; [6] J. Xiang et al. Int. J. Environ. Sci. Technol. 20 (2023) 1673-1688; [7] P. Kenchannavar and A. Surenjan Earth Environ. Sci. 1084 (2022) 012063.

**Table S12** – Total (BET), micropore (t-plot), and mesopore (BJH) surface area (m^2^ g^-1^), and pH_PZC_ of B9-B11, B9-B11^(BD)^, and B9-B11^(BD-TA)^.

| **Biochar** | **BET** | **t-plot** | **BJH** | **pH_PZC_** |
| --- | --- | --- | --- | --- |
| B9-B11 | 322 (37) | 239 (18) | 69 (10) | 10.2 (0.3) |
| B9-B11^(BD)^ | 389 (53) | 341 (29) | 36 (7) | 6.5 (0.3) |
| B9-B11^(BD-TA)^ | 460 (43) | 230 (21) | 170 (12) | 9.2 (0.1) |
| AC | 785 (59) | 467 (28) | 232 (34) | 9.9 (0.1) |

# Adsorption of dyes

**Figure S8** shows the trend of adsorption kinetics of B9-B11^(BD)^, B9-B11^(BD-TA)^, and AC towards MB and DY in ultrapure water solutions.

**(B)**

**(A)**

**Figure S8** – Removal percentage of B9-B11^(BD)^ (□), B9-B11^(BD-TA)^ (◆), and a commercial activated carbon (◇) towards 100 mg/L ultrapure water solutions of methylene blue (A) or direct yellow 50 (B), as a function of contact time. Concentration of biochars = 4000 mg/L; concentration of the commercial activated carbon = 400 mg/L.

**Figure S9** illustrates a representative example of the trend observed for MB concentrations during a breakthrough column experiment.


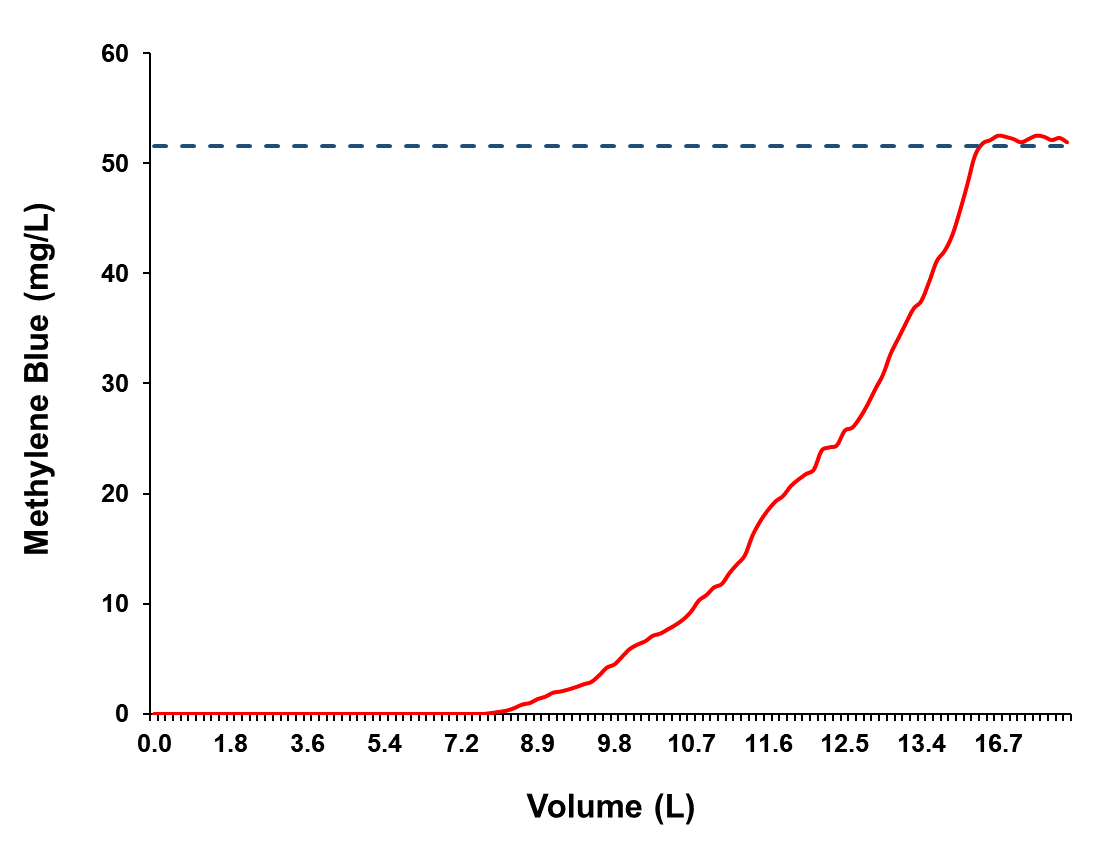


**Figure S9** – Methylene blue concentration trend during the filtration of approximately 18 L of wastewater fortified with 51.6 mg L^-1^ of methylene blue, through a column filled with 35 g of BC9-BC11^(BD-TA)^ at a flow rate of approximately 180 mL h^-1^. The dashed line indicates the initial concentration of methylene blue in the wastewater.
